# Research on automatic segmentation method of microseismic monitoring signal under low signal-to-noise ratio conditions

**DOI:** 10.1371/journal.pone.0358562

**Published:** 2026-09-18

**Authors:** Weijian Liu, Jianbo Li, Zhizeng Zhang, Zhenxia Yuan, Shuai Teng, Danqing Song, Mingrui Zhu, Wenlong Lv

**Affiliations:** 1 School of Smart Construction and Civil Engineering, Zhongyuan University of Technology, Zhengzhou, China; 2 School of Earth Science and Engineering, North China University of Water Resources and Electric Power, Zhengzhou, China; 3 School of Civil and Transportation Engineering, South China University of Technology, Guangzhou, China; China University of Mining and Technology, CHINA

## Abstract

To address the issues of reduced microseismic signal detection accuracy and difficulty in identifying event boundaries under low signal-to-noise ratio conditions, we propose SEU-Net, an automatic microseismic signal segmentation model that integrates a Squeeze-and-Excitation (SE) attention mechanism. This model embeds an SE module within the U-Net encoder, which enhances feature responses related to microseismic event boundaries through adaptive re-scaling of channel features. Simultaneously, it employs a temporal signal dimension expansion method to convert one-dimensional waveforms into an input format suitable for two-dimensional convolutional networks, thereby enabling end-to-end detection of microseismic event start and end times. Validation results using rock uniaxial compression acoustic emission test data show that SEU-Net achieves an overall segmentation accuracy of 98.97% on the test set, representing an improvement of approximately 1 percentage point over U-Net. Under a signal-to-noise ratio (SNR) of 5 dB, the proportions of samples with start and end point detection errors falling within the 0–5 range were 79.98% and 89.93%, respectively. Compared with EQTP, SEU-Net achieved an initial F1 score of 88.88% and an intersection-to-union ratio of 79.98%, which are higher than EQTP’s 86.81% and 76.69%, respectively. The error distribution and various evaluation metrics indicate that SEU-Net can maintain high accuracy in event boundary detection even under noise interference. Three-dimensional source localization based on the detection results shows that the spatial distribution of sources corresponding to SEU-Net is more concentrated and consistent with the actual fracture evolution process of the test specimen. This method provides technical support for the automatic detection of microseismic signals and source localization in complex rock mass engineering environments with low signal-to-noise ratios.

## 1. Introduction

Microseismic monitoring technology is a core component of early warning systems for rock mass engineering disasters. By continuously acquiring elastic wave signals generated by microfractures within the rock using sensors, and then processing and interpreting these signals, source location and energy calculation can be achieved, enabling prevention and early warning of rock mass disasters such as rockburstsand roof falls. The accuracy of signal acquisition directly determines the accuracy of source location and energy calculation, and is a crucial prerequisite for the reliability of the entire monitoring system [1,2].

Microseismic signals generated by coal and rock mass fracturing and surface well hydraulic fracturing are received by deployed microseismic sensors. The first step is filtering and denoising, as non-stationary noise significantly reduces the signal-to-noise ratio (SNR), affecting subsequent signal acquisition [3,4]. Zhao [5] proposed a coefficient attenuation method to address signal pollution caused by non-stationary noise, focusing on extracting effective signal components, thereby improving the SNR.The picking of microseismic events is mainly divided into two categories: manual picking and automatic picking. Classic automatic picking algorithms include the Short-Term Average/Long-Term Average (STA/LTA) method [6] and the Akaike Information Criterion (AIC) [7]. In response to the problem of insufficient accuracy of traditional methods under low signal-to-noise ratio conditions, many scholars have carried out improvement studies. In 2015, Joshua P. Jones et al. [8] proposed an improved STA/LTA detection method based on a dual-state hidden Markov model (HMM), which improved robustness while reducing parameter dependence; in 2017, Liu Xiaoming et al. [9] constructed a new feature function by introducing a weight factor K and combined it with the global maximum method to control the picking error under complex signal-to-noise ratio within the allowable range of engineering monitoring, but there is still a hidden danger of global extreme value misjudgment; Zhu Quanjie et al. [10] adopted a combined strategy of multi-threshold denoising and improved IAIC algorithm to significantly improve the signal-to-noise ratio and first arrival picking accuracy. The above-mentioned improved methods can only optimize the discrimination ability of a single feature. When faced with complex multi-wave interference and strong background noise scenarios, the stability and adaptability still have fundamental bottlenecks.

In recent years, deep learning has been extensively applied in microseismic signal processing. Early research focused on classification and effective signal recognition: in 2019, the deep CNN model constructed by Zhao Ming et al. [11] effectively resolved the high missed-detection rate of weak signals in massive datasets. In 2020, Wang Weibo et al. [12] further introduced 1D Convolutional Neural Networks (CNN) into microseismic event detection for hydraulic fracturing, achieving automatic feature extraction of 1D time-series waveforms and intelligent event-noise classification without the need to manually set thresholds. Under low-SNR conditions, it demonstrated feature mining potential and anti-noise robustness significantly superior to traditional time-frequency analysis methods. Subsequently, the research focus gradually shifted toward more refined point-wise semantic segmentation and precise onset arrival time picking. Zhao et al. [13] utilized a U-Net network for seismic phase segmentation, mitigating overfitting by introducing a Dropout layer, though its picking accuracy still had room for improvement. Guo et al. [14] applied an improved U-Net architecture (AE-PNet) to laboratory rock acoustic emission scenarios, verifying the effectiveness of U-Net-class models for small-sample, low-SNR microseismic signal picking. They pointed out the core limitations of existing models under strong noise interference: a lack of adaptive screening capability for effective features and a susceptibility to blurred signal boundary recognition. Cao Anye et al. [15] optimized a U-Net picking model specifically for mine microseismic scenarios, achieving accuracy and efficiency far exceeding traditional methods, yet issues regarding cross-domain adaptation and picking under low-SNR conditions remained unresolved. To overcome complex noise interference, some scholars began introducing attention mechanisms into their models. For instance, Jiao Mingruo et al. [16] proposed a microseismic picking method integrating Gated Recurrent Units (GRU) and self-attention, demonstrating high accuracy and noise resistance in small-sample scenarios. However, the processing workflow of such methods is relatively cumbersome, and the model’s cross-domain generalization capability and engineering deployment convenience require further optimization.A robust source localization model underpins both the dynamic monitoring of engineering projects and the mitigation of disaster risks throughout the construction lifecycle [17,18].

In summary, significant progress has been made in the intelligent acquisition of microseismic signals, but there are obvious technical shortcomings in low signal-to-noise ratio and strong background interference environments [19–21]. Existing convolutional networks are insufficient to meet the multi-scale feature extraction requirements of time-series signals. Traditional one-dimensional convolution is inadequate in representing signal abrupt boundary changes, leading to acquisition errors. Standard U-Net lacks an adaptive feature selection mechanism for the channel dimension, making it difficult to effectively distinguish between valid signal and noise features in noisy environments, resulting in a sharp decline in acquisition accuracy and robustness under low signal-to-noise ratio conditions.

To address the aforementioned challenges, this paper proposes an automatic microseismic signal segmentation method based on the Squeeze-and-Excitation (SE) attention mechanism [22] from the perspective of temporal feature selection and boundary reinforcement modeling. This method explicitly models the dependencies between channels on the basis of the U-Net architecture, adaptively enhances the channel weights related to the abrupt changes in microseismic signals, and suppresses the background noise response. To address the mismatch between one-dimensional microseismic signals and the standard U-Net input format, this paper performs dimensional expansion in the data preprocessing stage. In TensorFlow, the one-dimensional microseismic signal is expanded into a three-dimensional tensor format using tf.newaxis, so that the data meets the network input requirements. This achieves end-to-end accurate picking of microseismic events. Based on laboratory rock uniaxial compression and acoustic emission experiments, the picking accuracy of the proposed method is verified, laying the foundation for the application of this method in practical engineering scenarios.

## 2. Model implementation and data processing

### 2.1. Overall architecture and implementation principles of SEU-Net

**Fig 1:**
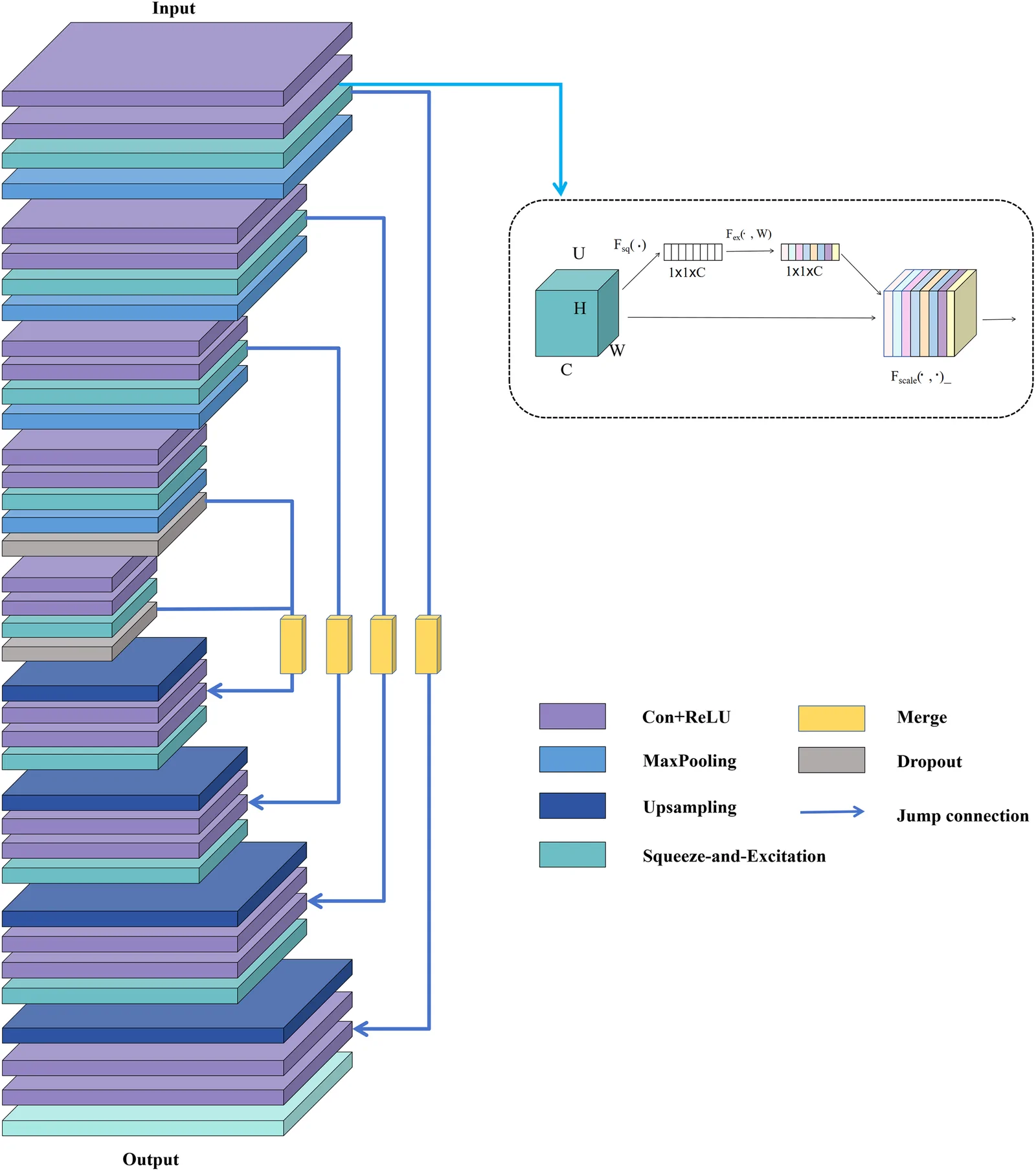
Network architecture.

The proposed SEU-Net model aims to achieve end-to-end automatic segmentation and accurate start-end time acquisition of microseismic signals, and its main architecture is shown in Fig 1. This model follows the classic encoder-decoder design paradigm and innovatively integrates a Squeeze-and-Excitation attention mechanism and a multi-scale skip connection strategy. Specifically, the encoder is responsible for extracting high-order temporal features through layer-by-layer convolution and downsampling; the decoder gradually recovers the temporal resolution of the signal through upsampling and fuses shallow location information using skip connections. The dashed box in the figure illustrates the adaptive recalibration process of the feature channel weights in the SE module, and the yellow module represents the feature concatenation operation, which aims to alleviate the gradient vanishing problem in deep networks and promote the effective fusion of multi-scale information.For detailed information on the input/output dimensions and convolutional kernel configurations for each layer of the network, see Table 1.

**Table 1:** Layer-wise parameters.

Layer stage	Operation	Input size	Output size	Kernel size
Input	Input layer	–	1024 × 1 × 1	–
Encoder 1	Conv2D+ReLU × 2	1024 × 1 × 1	1024 × 1 × 64	(3,1)
	SE Block	1024 × 1 × 64	1024 × 1 × 64	–
	MaxPooling2D	1024 × 1 × 64	512 × 1 × 64	(2,1)
Encoder 2	Conv2D+ReLU × 2	512 × 1 × 64	512 × 1 × 128	(3,1)
	SE Block	512 × 1 × 128	512 × 1 × 128	–
	MaxPooling2D	512 × 1 × 128	256 × 1 × 128	(2,1)
Encoder 3	Conv2D+ReLU × 2	256 × 1 × 128	256 × 1 × 256	(3,1)
	SE Block	256 × 1 × 256	256 × 1 × 256	–
	MaxPooling2D	256 × 1 × 256	128 × 1 × 256	(2,1)
Encoder 4	Conv2D+ReLU × 2	128 × 1 × 256	128 × 1 × 512	(3,1)
	SE Block	128 × 1 × 512	128 × 1 × 512	–
	Dropout	128 × 1 × 512	128 × 1 × 512	–
	MaxPooling2D	128 × 1 × 512	64 × 1 × 512	(2,1)
Bottleneck	Conv2D+ReLU × 2	64 × 1 × 512	64 × 1 × 1024	(3,1)
	SE Block	64 × 1 × 1024	64 × 1 × 1024	–
	Dropout	64 × 1 × 1024	64 × 1 × 1024	–
Decoder 1	UpSampling2D+Conv2D + ReLU	64 × 1 × 1024	128 × 1 × 512	(2,1)
	Concatenate	128 × 1 × 512 + 128 × 1 × 512	128 × 1 × 1024	–
	Conv2D+ReLU × 2	128 × 1 × 1024	128 × 1 × 512	(3,1)
	SE Block	128 × 1 × 512	128 × 1 × 512	–
Decoder 2	UpSampling2D+Conv2D + ReLU	128 × 1 × 512	256 × 1 × 256	(2,1)
	Concatenate	256 × 1 × 256 + 256 × 1 × 256	256 × 1 × 512	–
	Conv2D+ReLU × 2	256 × 1 × 512	256 × 1 × 256	(3,1)
	SE Block	256 × 1 × 256	256 × 1 × 256	–
Decoder 3	UpSampling2D+Conv2D + ReLU	256 × 1 × 256	512 × 1 × 128	(2,1)
	Concatenate	512 × 1 × 128 + 512 × 1 × 128	512 × 1 × 256	–
	Conv2D+ReLU × 2	512 × 1 × 256	512 × 1 × 128	(3,1)
	SE Block	512 × 1 × 128	512 × 1 × 128	–
Decoder 4	UpSampling2D+Conv2D + ReLU	512 × 1 × 128	1024 × 1 × 64	(2, 1)
	Concatenate	1024 × 1 × 64 + 1024 × 1 × 64	1024 × 1 × 128	–
	Conv2D+ReLU × 2	1024 × 1 × 128	1024 × 1 × 64	(3,1)
	SE Block	1024 × 1 × 64	1024 × 1 × 64	–
Output	Conv2D+ReLU	1024 × 1 × 64	1024 × 1 × 2	(3,1)
	Conv2D+Sigmoid	1024 × 1 × 2	1024 × 1 × 1	(1,1)

#### 2.1.1. Encoder module.

The encoder consists of multiple cascaded coding units, performing feature extraction and dimensionality compression. Inputting microseismic data, the initial convolutional layer has 64 channels, which increases exponentially with network depth (64 → 128 → … → 1024), while the temporal dimension is halved sequentially through (2,1) max pooling. Each coding unit contains two 3 × 1 convolutional layers sliding along the time axis, followed by batch normalization and a ReLU activation function to capture local waveform features, stabilize the training process, and accelerate convergence.

To address the challenge of equal processing of feature channels in traditional convolutional operations, this paper embeds an SE attention module after each convolutional block. Furthermore, Dropout layers with a dropout rate of 0.5 are added to layers 4 and 5 of the deep network to enhance regularization. The convolutional operation of layer l is as follows:


$$\begin{array}{c} X_{l}=\delta \left(W_{l}*X_{l-\text{1}}+b_{l}\right) \end{array}$$ (1)

where $X_{l}$ represents the output feature map of the l-th layer, $X_{l-\text{1}}$ denotes the input feature map of the l−1-th layer, $W_{l}$ is the weight matrix, $b_{l}$ is the bias term, * indicates the convolution operation, and δ is the ReLU activation function.

#### 2.1.2. SE attention mechanism.

The core of the SE module lies in explicitly modeling the dependencies between feature channels, achieving “adaptive gating” of features. Its computation process includes three key steps:

Squeeze: Global average pooling is performed on the feature map to compress the temporal information into a channel descriptor, realizing the aggregation of global receptive-field features:


$$\begin{array}{c} Z_{c}=\frac{\text{1}}{T\times W}\sum_{t=\text{1}}^{T}\sum_{w=\text{1}}^{W}X_{c}\left(t,w\right) \end{array}$$ (2)

where $Z_{c}$ denotes the global aggregated feature of the c-th channel, T represents the number of time steps of the feature map, and $X_{c}\left(t,w\right)$ is the feature value of the c-th channel at time step t and width position w. In this study, an anisotropic convolution kernel of size (3,1) slides exclusively along the time axis, resulting in W=1 for all feature maps.

Excitation: A bottleneck structure comprising two fully connected (FC) layers is used to learn the nonlinear relationships among channels and output a normalized channel‑weight vector$S=[S_{\text{1}},S_{\text{2}}\;,\ldots ,S_{\text{c}}]:$


$$\begin{array}{c} S=\sigma \left(W_{\text{2}}\delta \left(W_{\text{1}}Z\right)\right) \end{array}$$ (3)

where δ denotes the ReLU activation function, and σ represents the Sigmoid activation function. $W_{\text{1}}$ and $W_{\text{2}}$ are learnable weight matrices. This process essentially acts as a feature‑gating mechanism.

Reweight: The normalized weight $S_{c}$ for the c-th channelis is multiplied channel-by-channel with the original two‑dimensional feature map $X_{\text{c}}\left(t,w\right)$ to accomplish feature enhancement:


$$\begin{array}{c} {\tilde{X}}_{c}\left(t,w\right)=S_{c}\cdot X_{\text{c}}\left(t,w\right) \end{array}$$ (4)

Benefiting from adaptive channel weighting, the probability distribution output by the model exhibits distinct rising and falling edges at the onset and termination of seismic events, which can effectively reduce arrival‑time picking errors.

#### 2.1.3. Decoder module.

Transposed convolutions of size (2,1) upsample the feature maps, progressively restoring temporal resolution. Shallow features from the encoder are concatenated with the upsampled deep features from the decoder. This compensates for spatial location information lost due to downsampling and improves the model’s accuracy in locating microseismic event boundaries by fusing shallow boundary details with deep semantic features.

#### 2.1.4. Output layer and loss function.

For an input time series of length L=1024, the model reduces the feature dimension to 1 via a 1 × 1 convolution in the output layer and maps the output to the interval [0,1] using the Sigmoid activation function, generating a probability sequence $P=\{p_{\text{1}},p_{\text{2}},...,p_{\text{1}\text{0}\text{2}\text{4}}\}$ of the same length as the input waveform.$p_{i}\in [\text{0},\text{1}]$ represents the probability that the i-th time step belongs to a “microseismic event.” The true labels $Y\in \{\text{0},\text{1}{\}}^{\text{1}\text{0}\text{2}\text{4}\times \text{1}}$ have the same dimension as P, and $y_{i}\in \{\text{0},\text{1}\}$ is the binary label for the i-th time step. A value of 1 indicates a valid time step, while 0 indicates noise.

During training, the Weighted Binary Cross-Entropy (Weighted BCE) loss is used to calculate the loss for the predicted probability $p_{i}$ and the label $y_{i}$ point by point along the time dimension. The final loss is obtained by averaging over all time steps:


$$\begin{array}{c} \begin{array}{c} L=-\frac{\text{1}}{n}\sum_{i=\text{1}}^{n}\left[{w_{pos}\cdot y}_{i}log\left(p_{i}\right)+\left(\text{1}-y_{i}\right)log\left(\text{1}-p_{i}\right)\right] \end{array} \end{array}$$ (5)

where n = 1024 is the total number of sampling points, and wpos is the loss weight for positive microseismic samples (event time steps); this weight is calculated based on the inverse ratio of the number of positive and negative sampling points in the training set. To address the issue of significant disparities in the distribution of lithological samples in the dataset—which can easily lead to cross-lithological generalization bias in the model—this paper further introduces a lithological-level sample weighting mechanism. This mechanism assigns higher loss weights to lithological samples with a lower proportion, thereby increasing the contribution of underrepresented lithologies to parameter updates and mitigating the learning bias toward dominant lithological classes.

The final total training loss is:


$$\begin{array}{c} L_{total}=\frac{\text{1}}{N_{total}}\sum_{k=\text{1}}^{K}\;\sum_{j=\text{1}}^{N_{k}}\;w_{k}\cdot L_{WBCE}^{\left(k,j\right)} \end{array}$$ (6)

where: K is the total number of lithological classes in the dataset; ${\text{N}}_{\text{k}}$ is the number of training samples corresponding to the k-th lithological class; ${\text{N}}_{\text{total}}$ is the total number of samples in the training set; ${\text{L}}_{\text{WBCE}}^{\left(\text{k},\text{j}\right)}$ is the point-wise weighted binary cross-entropy loss for the j-th sample in lithological class k, i.e., the result of Equation (5); ${\text{w}}_{\text{k}}$ is the sample loss weight for lithological class k, calculated based on the inverse frequency of lithological samples in the training set: ${\text{w}}_{\text{k}}=\frac{{\text{N}}_{\text{total}}}{\text{K}\cdot {\text{N}}_{\text{k}}}$, This dual-weighting mechanism simultaneously constrains category balance at the point level and lithology balance at the sample level, enhancing the model’s cross-lithology generalization ability while ensuring detection accuracy.

### 2.2. Data preprocessing

#### 2.2.1. Dataset construction.

The dataset is a compilation of uniaxial compression acoustic emission experiments of rocks conducted by our research group [23] The experimental specimens include three types: coal, cement mortar, and sandstone. Continuous elastic wave signals during the compression fracture process were collected using an acoustic emission system. This paper adjusts the original data from our research group: the length of the input event time series is 1024, and the arrival and end times of the microseismic events are manually marked. Finally, a total of 8100 valid microseismic waveform samples were compiled, including 1242 coal fracture signals, 6528 cement mortar signals, and 330 sandstone signals. This dataset covers the frequency band characteristics and energy differences of different lithologies during fracture, ensuring the diversity of model training data.To mitigate the impact of data imbalance on model generalization, we employed a weighted binary cross-entropy loss, assigning higher weights to the underrepresented sandstone and coal samples to reduce the model’s bias toward the cement mortar class.

#### 2.2.2. Preprocessing.

Due to issues such as inconsistent duration and baseline drift in the original microseismic signals, the following standardized preprocessing procedure was implemented to meet the input specifications of deep learning models:

Amplitude normalization: In order to eliminate the difference in acquisition gain and accelerate model convergence, the data is demeaned to eliminate the DC component, and the maximum absolute value normalization is used to map the signal amplitude to a uniform scale.

Dimension Adaptation: The standard U-Net architecture includes a large number of two-dimensional convolution and pooling operations and is natively suited for image data. It cannot directly process one-dimensional microseismic time-series signals. In this paper, we use the `tf.newaxis` interface in the TensorFlow framework to extend the dimensionality axis, transforming a one-dimensional sequence x ∈ ℝ1024 of length L = 1024 into a three-dimensional tensor format X ∈ ℝ1024 × 1 × 1 (i.e., with a time step of 1024, a width of 1, and one channel). An anisotropic convolution kernel of size (3,1) is used to perform sliding convolution along the time axis.

#### 2.2.3. Semantic label generation and dataset partitioning.

This paper transforms the signal acquisition task into a point-by-point binary classification problem. Based on manually labeled start and end times, a mask vector of the same length as the input waveform is constructed. All labels were independently annotated by two researchers with more than 2 years of experience in microseismic signal processing. For inconsistent labeling results, a third senior researcher was invited for arbitration to ensure the accuracy of the ground truth.

**Fig 2:**
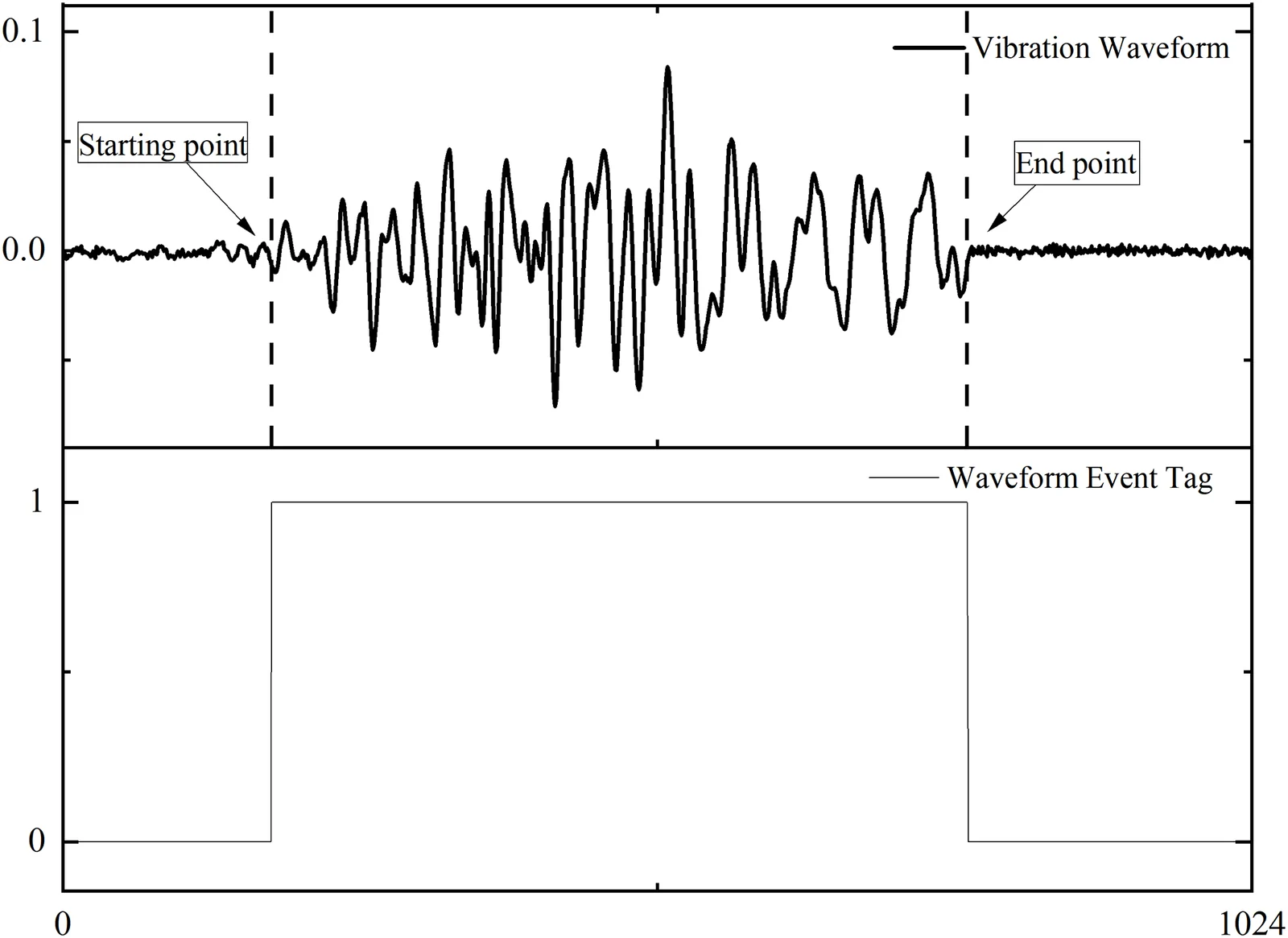
SEU-Net label annotation diagram.

The time period in which the microseismic event exists is marked as 1, and background noise and filled regions are marked as 0, forming a supervision label in the form of “0-1-0”. The schematic relationship between the input waveform and the corresponding label is shown in Fig 2.

To ensure an objective and reliable performance evaluation and eliminate the risk of data leakage, the dataset is partitioned strictly in accordance with the independence of experimental specimens, instead of pure random splitting. Specifically, all waveform samples derived from the same rock specimen and the same loading stage are classified into the same subset, so that the data sources of the training set, validation set and test set are completely independent of each other. The overall volume ratio of the training set, validation set and test set is controlled at 8:1:1.

## 3. Results and discussion

### 3.1. Model convergence analysis

**Fig 3:**
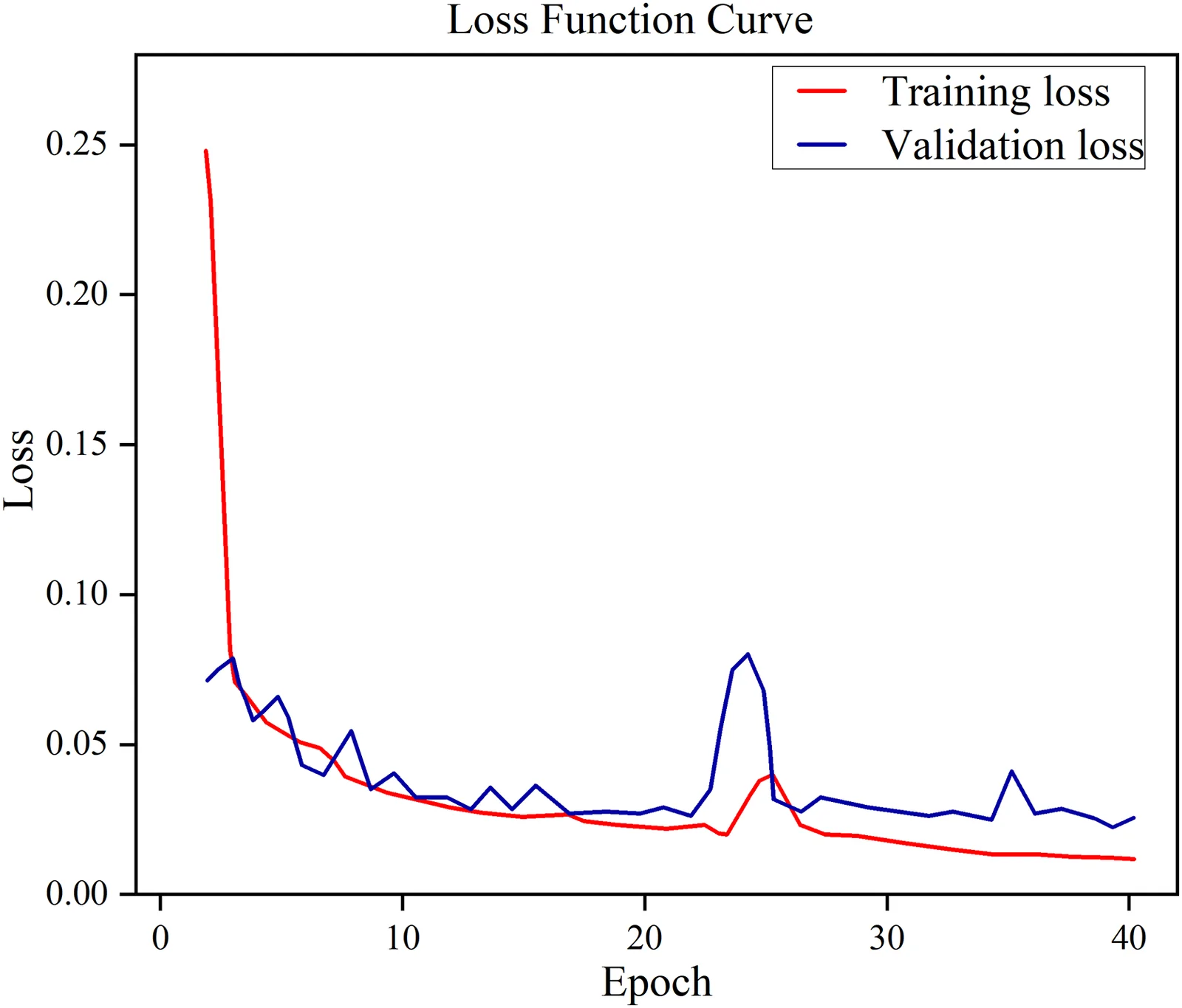
Loss function curve.

**Fig 4:**
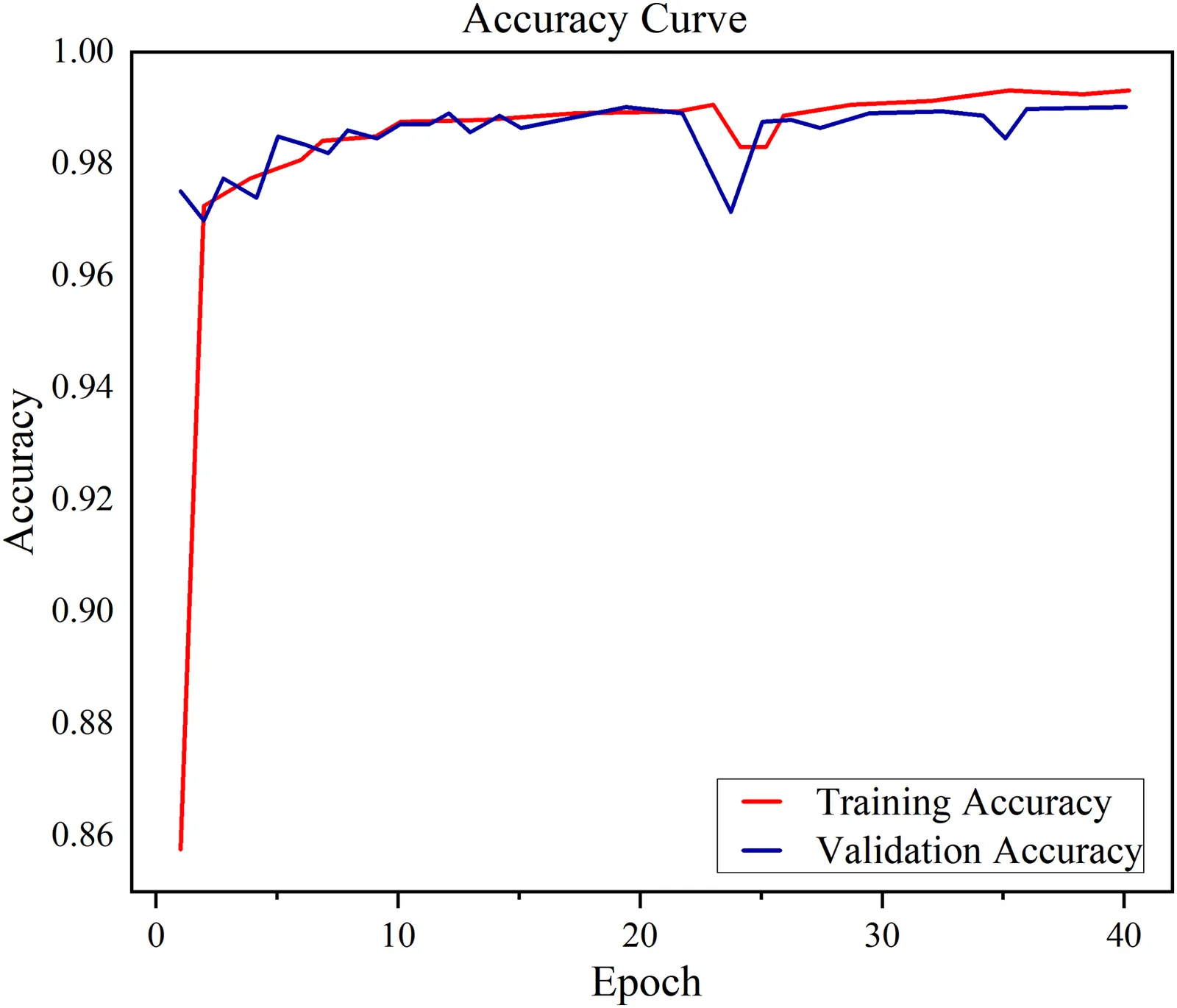
Accuracy curve.

This paper builds and trains the SEU-Net model based on the TensorFlow deep learning framework. An NVIDIA GeForce GTX 1650 dedicated graphics card (4GB VRAM) was selected for training. To ensure the stability and efficiency of model training, after multiple parameter optimizations, a batch size of 32 and an initial learning rate of 0.001 were adopted. The Adam optimizer was used for gradient updates, yielding optimal results. The number of iterations was set to 40. The loss function and accuracy curves of the model during training are shown in Figs 3 and 4. In the loss curve of Fig 3, the training set loss decreases rapidly from 0.25, and within the first 20 iterations, the validation set loss decreases synchronously with the training loss. Following minor oscillations around the 23rd epoch, the curves converge and stabilize, demonstrating the model’s excellent generalization capability and robustness.As indicated by the accuracy curve in Fig 4, the training set accuracy increases from 85.4% to 98.3% within the first 10 epochs, demonstrating a high learning efficiency. From the 25th epoch onwards, the accuracy curve begins to stabilize, ultimately settling, at approximately 98.97% with minimal fluctuations. This indicates that the model has reached an optimal convergence state by the 40th epoch.

### 3.2. Performance testing under noise-free and noisy conditions

This paper presents a comparative experiment under noise-free and low signal-to-noise ratio (SNR = 5 dB) environments. The experiment defines a picking error occurring outside the first 50 samples as a faulty picking; the percentage of samples with errors within the first 5 samples is defined as the picking accuracy.Experimental data were sampled at a frequency of 3 MHz, corresponding to a sampling interval of approximately 0.33 μs. An error of 5 sampling points corresponds to a time deviation of approximately 1.67 μs. This magnitude falls within the permissible error range for engineering microseismic monitoring and source location and will not significantly affect the accuracy of subsequent source location results. SNR = 5 dB is selected to represent a complex scenario in microseismic monitoring. Under these conditions, the P-wave picking points of the microseismic signal are not obvious in the time domain (as shown in Fig 7).

**Fig 5:**
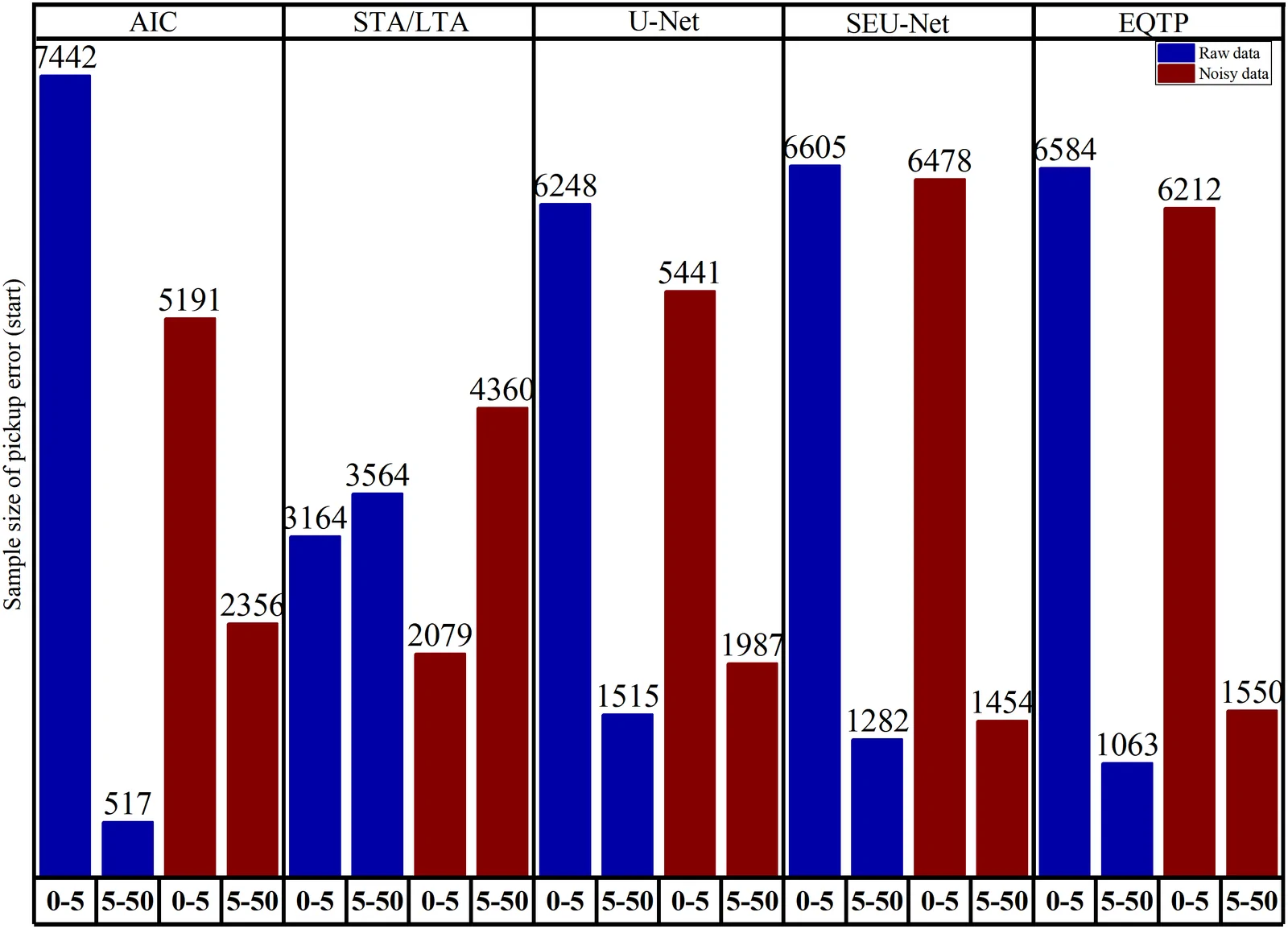
Sample size of picking error (start).

**Fig 6:**
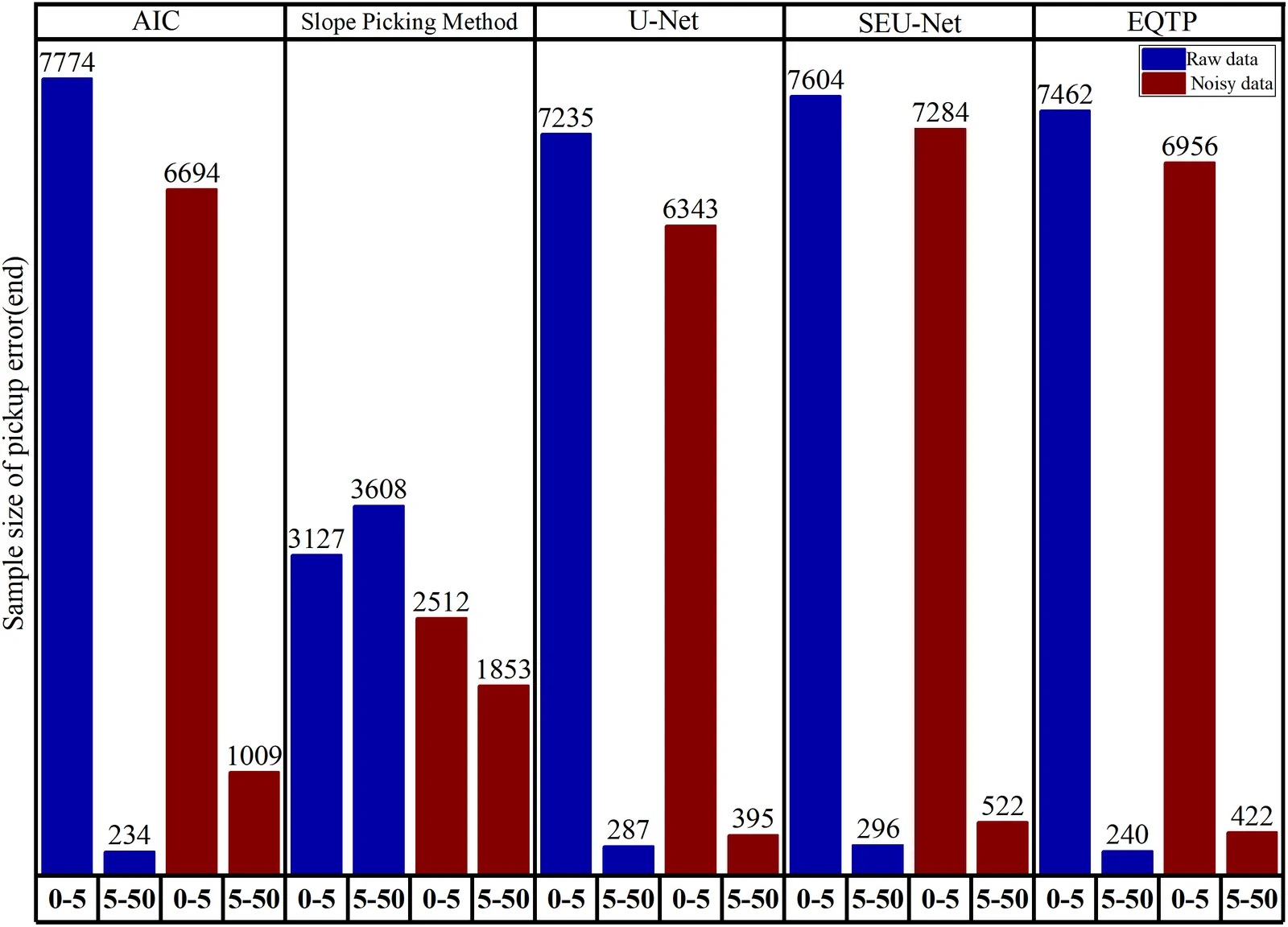
Sample size of picking error (end).

Using the same dataset, we performed signal extraction on both the original waveforms and noise-added waveforms using traditional methods such as AIC, time-window energy eigenvalues, and the slope-based extraction method, as well as deep learning models including U-Net, SEU-Net, and the advanced model EQTP [24], which was proposed in recent years. To systematically analyze the impact of attention mechanisms on improving extraction performance, this paper quantifies the performance of each method across the following metrics: the distribution of detection errors, the proportion of samples in each error interval, the false detection rate, and the average error. The detailed results are shown in Figs 5 and 6 and Table 2 below.

**Table 2:** Comparison table of accuracy before and after adding noise.

Method	Waveform Picking Error Point		Proportion/%		MAE/μs		RMSE/μs	
			Noiseless	Add noise	Noiseless	Add noise	Noiseless	Add noise
U-Net	Initial point	0 ~ 5	77.14	67.17	4.44	6.96	10.97	15.12
		5 ~ 50	18.70	24.53				
		50+	4.16	8.30				
SEU-Net		0 ~ 5	81.54	79.98	3.45	3.35	8.92	8.21
		5 ~ 50	14.59	17.95				
		50+	2.63	3.31				
EQTP		0 ~ 5	81.28	76.69	4.68	4.48	12.30	10.99
		5 ~ 50	18.6	19.14				
		50+	0.65	4.17				
U-Net	End point	0 ~ 5	89.32	78.31	4.64	9.50	13.49	20.61
		5 ~ 50	3.54	4.88				
		50+	7.14	16.81				
SEU-Net		0 ~ 5	93.88	89.93	2.35	3.15	8.09	9.84
		5 ~ 50	3.65	6.44				
		50+	2.47	3.63				
EQTP		0 ~ 5	94.59	87.11	3.49	5.65	11.22	15.09
		5 ~ 50	2.96	5.21				
		50+	2.44	7.68				

Figs 5 and 6 show the sample distribution of different methods in different error intervals. As can be seen from the figure, the STA/LTA method has low picking accuracy under both noise-free and noisy conditions, indicating its weakest anti-interference ability. The slope method shows some adaptability in picking the endpoint, but its overall picking effect is poor. The AIC method performs well in a noise-free environment, but after adding noise, the number of samples in the 0–5 interval drops sharply from a high value to 5191 and 6694, indicating that the picking accuracy of this method is significantly affected under low signal-to-noise ratio conditions.

Table 2 quantifies the differences in picking accuracy between U-Net, EQTP, and the proposed SEU-Net model. U-Net’s picking accuracy was 77.14% (starting point) and 89.32% (ending point) without noise, decreasing to 67.17% and 78.31% after adding noise, a drop of approximately 10 percentage points, indicating weak noise resistance. EQTP’s picking accuracy was 81.28% (starting point) and 94.59% (ending point) without noise, decreasing to 76.69% and 89.11% after adding noise, a drop of approximately 5 percentage points, indicating a performance decline after adding noise and weak noise resistance. The proposed model achieved picking accuracy of 81.54% at the starting point and 93.88% at the ending point without noise. After adding noise, the starting point accuracy remained at 79.97%, while the ending point accuracy only decreased by about 4 percentage points, indicating that noise had a relatively small impact. This demonstrates that the SE attention mechanism suppresses redundant noise channels and focuses the network on subtle waveform abrupt changes.

In terms of absolute picking error, SEU-Net also achieves the lowest MAE and RMSE under both noiseless and noisy conditions (Table 2). For start-point detection, SEU-Net achieved MAE values of 3.45 μs and 3.35 μs under noise-free and 5 dB noise conditions, respectively, significantly outperforming U-Net’s 4.44 μs and 6.96 μs and EQTP’s 4.68 μs and 4.48 μs; SEU-Net’s RMSE was 8.92 μs and 8.21 μs, respectively, which is also notably lower than U-Net’s 10.97 μs and 15.12 μs. For endpoint detection, in particular, the RMSE values of 8.09 μs and 9.84 μs are significantly better than those of U-Net (13.49 μs and 20.61 μs) and EQTP (11.22 μs and 15.09 μs). The ratio of RMSE to MAE for SEU-Net is close to 1.0, indicating that its error distribution is more concentrated around the mean and contains fewer outliers with large deviations. This advantage also demonstrates the SE module’s effective suppression of noise-induced false detections.

Table 3 lists the precision, recall, F1 score, and precision-recall ratio for each model under noise-free and 5 dB noise conditions, respectively. As shown in Table 3, EQTP achieves higher precision under noise-free conditions, but SEU-Net demonstrates greater robustness in noisy environments. Under 5 dB noise, the F1 score of SEU-Net at the start point was 88.88%, a decrease of only 0.95 percentage points compared with the noise-free state—far lower than the 6.75 point decline observed in U-Net and the decline in the EQTP model. Crucially, the misclassification rate of SEU-Net at the endpoint is only 3.63%, versus 7.68% for EQTP. This further validates that the attention mechanism of SEU-Net can effectively suppress noise interference while preserving key waveform features, thereby significantly improving recognition accuracy and noise resistance.

**Table 3:** Comparison of segmentation evaluation metrics for microseismic event start and end points.

Method	Waveform Picking	Noise condition	Precision	Recall	F1 score	IoU
U-Net	Initial point	Noiseless	80.50%	94.87%	87.11%	77.14%
		Add noise	73.25%	89.00%	80.36%	67.17%
SEU-Net		Noiseless	83.67%	96.86%	89.83%	81.54%
		Add noise	81.67%	96.04%	88.88%	79.98%
EQTP		Noiseless	83.57%	96.94%	89.67%	81.28%
		Add noise	80.32%	94.45%	86.81%	76.69%
U-Net	End point	Noiseless	96.19%	92.60%	94.36%	89.32%
		Add noise	94.14%	82.22%	87.77%	78.31%
SEU-Net		Noiseless	96.25%	97.44%	96.84%	93.88%
		Add noise	93.32%	96.12%	94.70%	89.93%
EQTP		Noiseless	96.63%	97.82%	97.22%	94.59%
		Add noise	91.76%	94.51%	93.11%	87.11%

We also added separate test accuracy statistics for the three lithologies. The results show that the picking accuracy of the model on sandstone samples still reaches 96.2%, verifying that the model still maintains good generalization performance on minority lithological samples.

In summary, quantitative comparisons show that the proposed SEU-Net not only outperforms the traditional U-Net baseline model but also demonstrates exceptional performance when compared to the state-of-the-art EQTP model. Crucially, under low signal-to-noise ratio conditions, it maintains excellent boundary accuracy while achieving a lower false positive rate.

**Fig 7:**
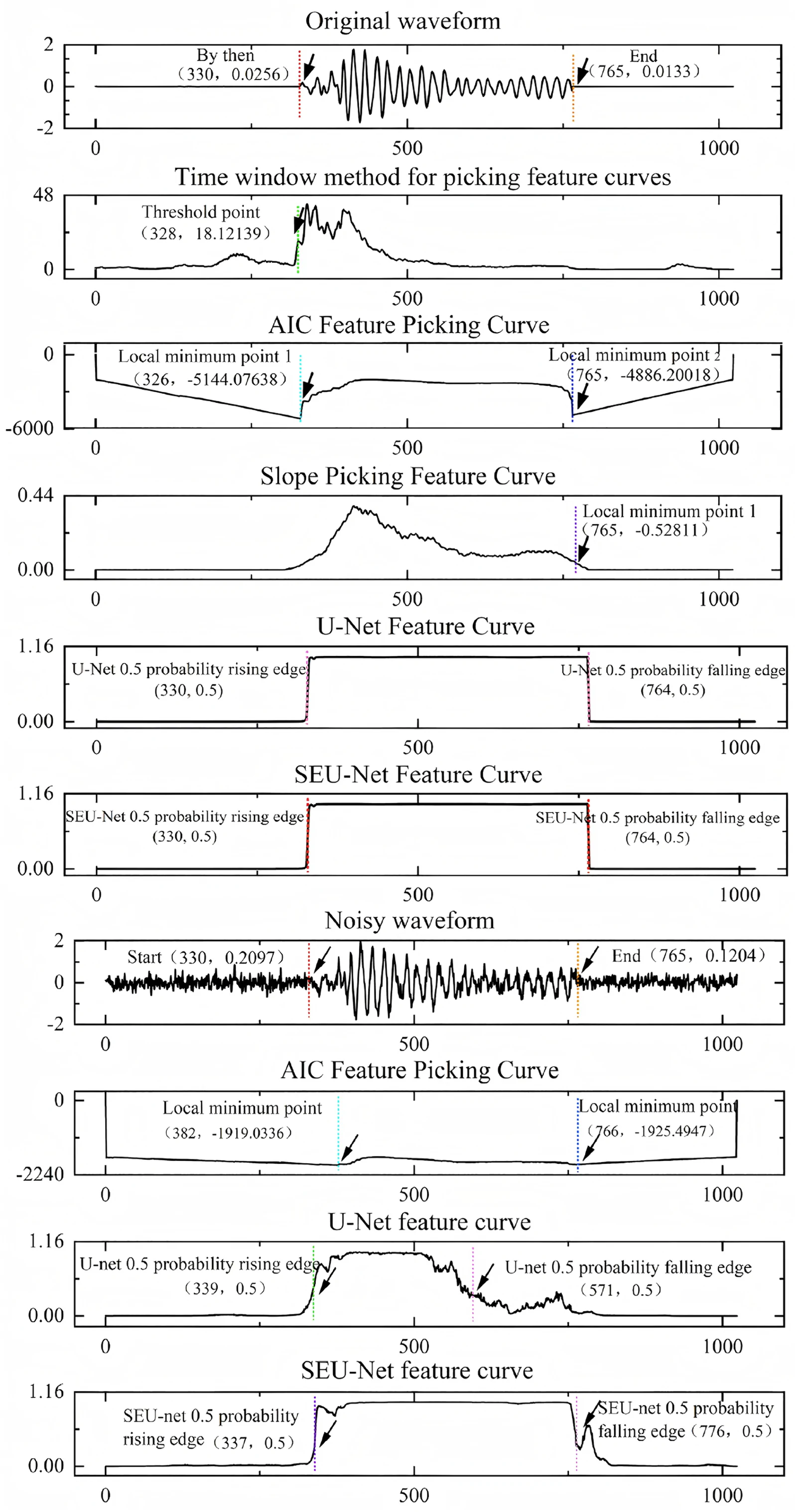
Comparison of feature maps extracted from original and noise-added waveform.

Fig 7 shows a comparison of the picking features of different methods under the original waveform and the noisy waveform. The noisy test group demonstrates three representative methods. In the original waveform, the picking point features of the microseismic signal are obvious, and the global minimum point of the AIC function curve can accurately correspond to the start and end points of the signal. The probability envelopes output by U-Net and SEU-Net also exhibit a rectangular label shape with steep edges, indicating that both can accurately determine the event boundary, demonstrating that in noise-free picking, both the standard model and the model in this paper can achieve accurate picking. However, under strong noise interference, the picking capabilities of each method show significant differences. The feature curve of the AIC method becomes blurred due to noise, resulting in serious errors in its first arrival picking point. The U-Net model can identify the first arrival time, but its feature extraction capability is weak at the end of the picking point. Its output probability response curve exhibits severe jitter at the end of the signal, leading to premature decay and failing to fully cover the effective range of the microseismic event. Compared to the proposed SEU-Net model, this paper demonstrates stronger noise robustness, maintaining the relative integrity and stability of the probability rectangle curve even under equally strong noise conditions. It accurately captures not only the initial arrival point but also pinpoints the final arrival point to its true location. However, the probability curve exhibits jitter later on. The picked points are within acceptable limits. Further results validate that the introduction of the SE module effectively suppresses redundant noise interference, significantly enhancing the model’s feature recognition and event picking capabilities in low signal-to-noise ratio environments.

## 4. Laboratory validation

**Fig 8:**
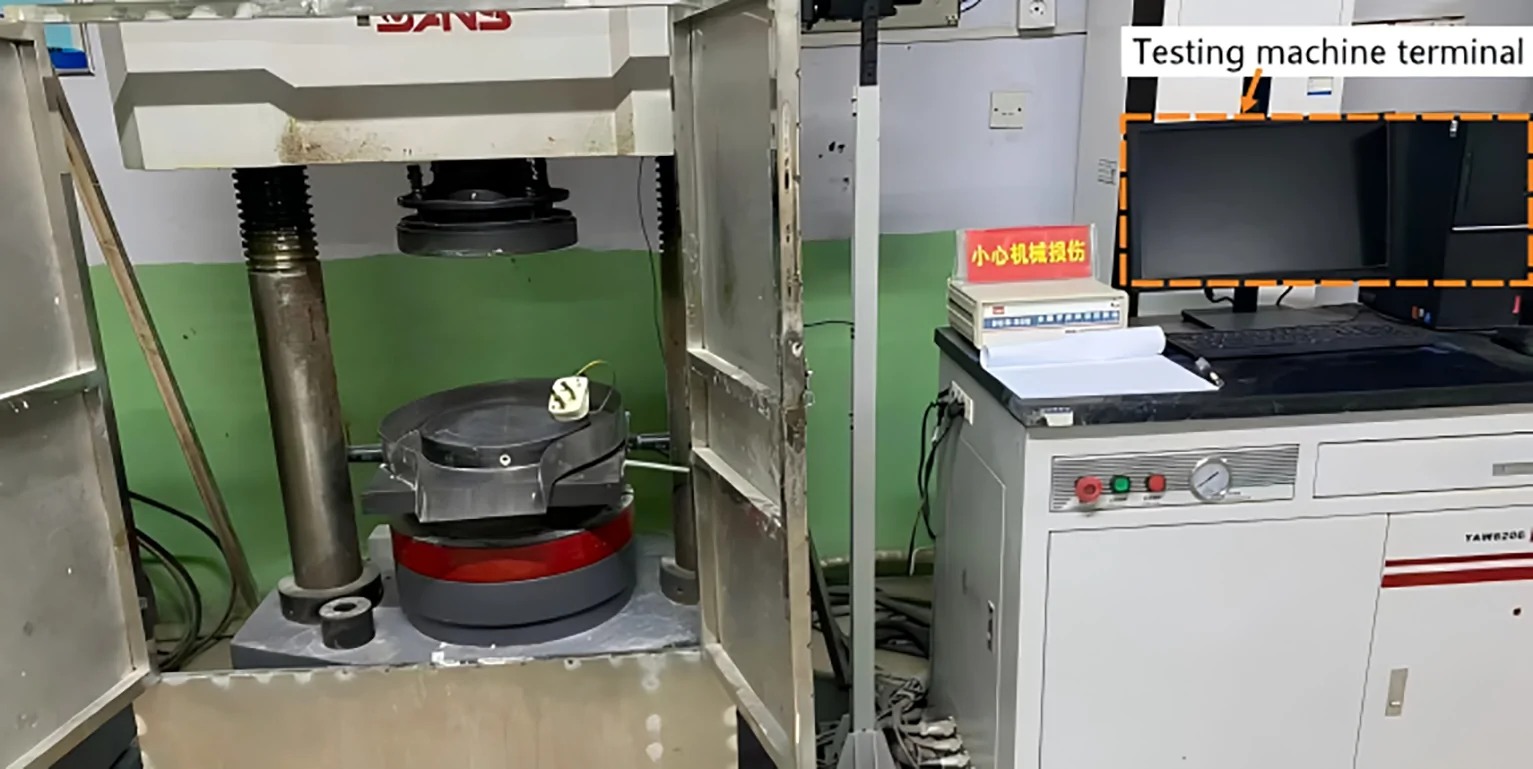
Microcomputer-controlled electro-hydraulic servo pressure testing machine.

**Fig 9:**
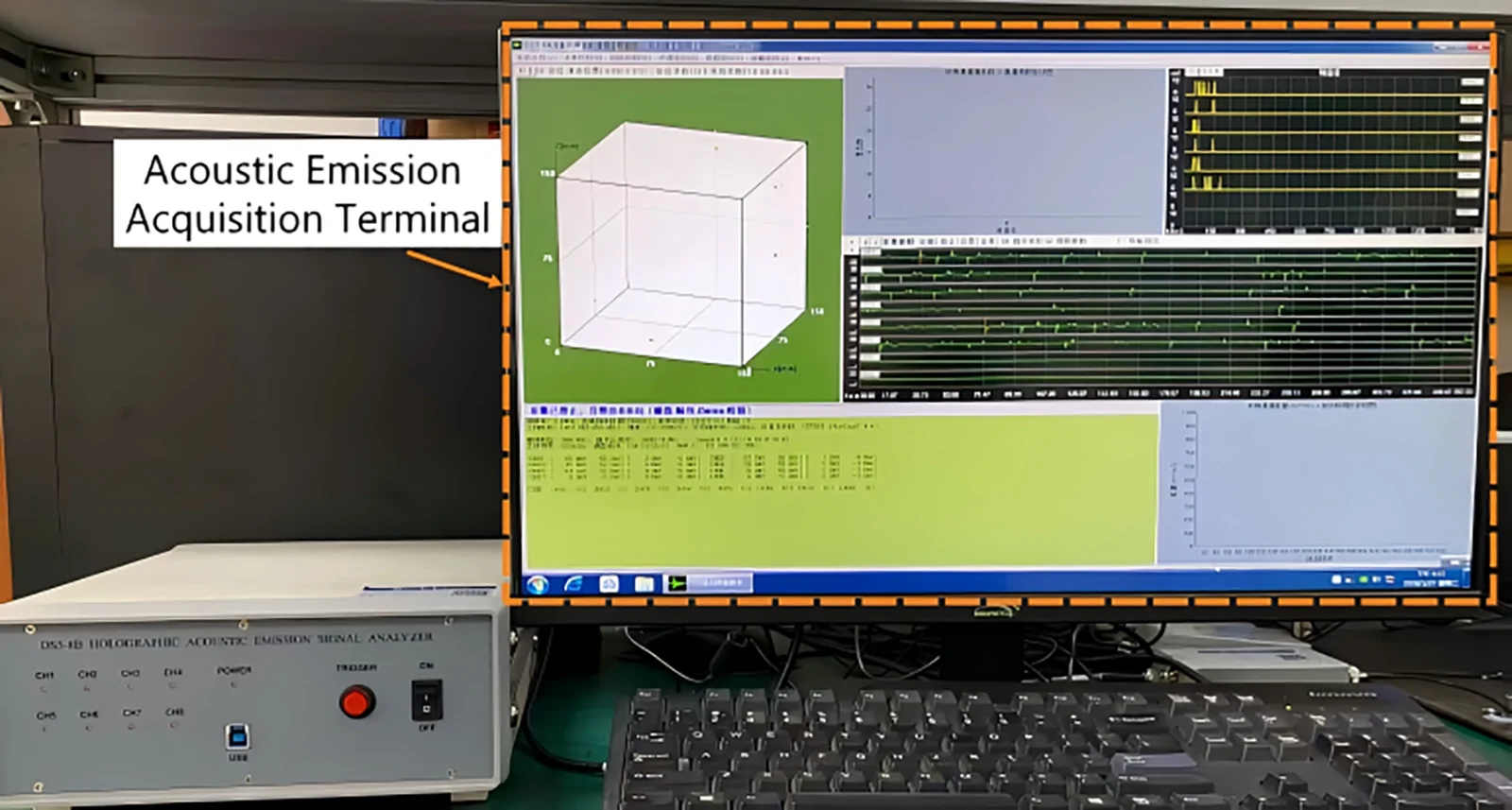
Channel acoustic emission instrument.

**Fig 10:**
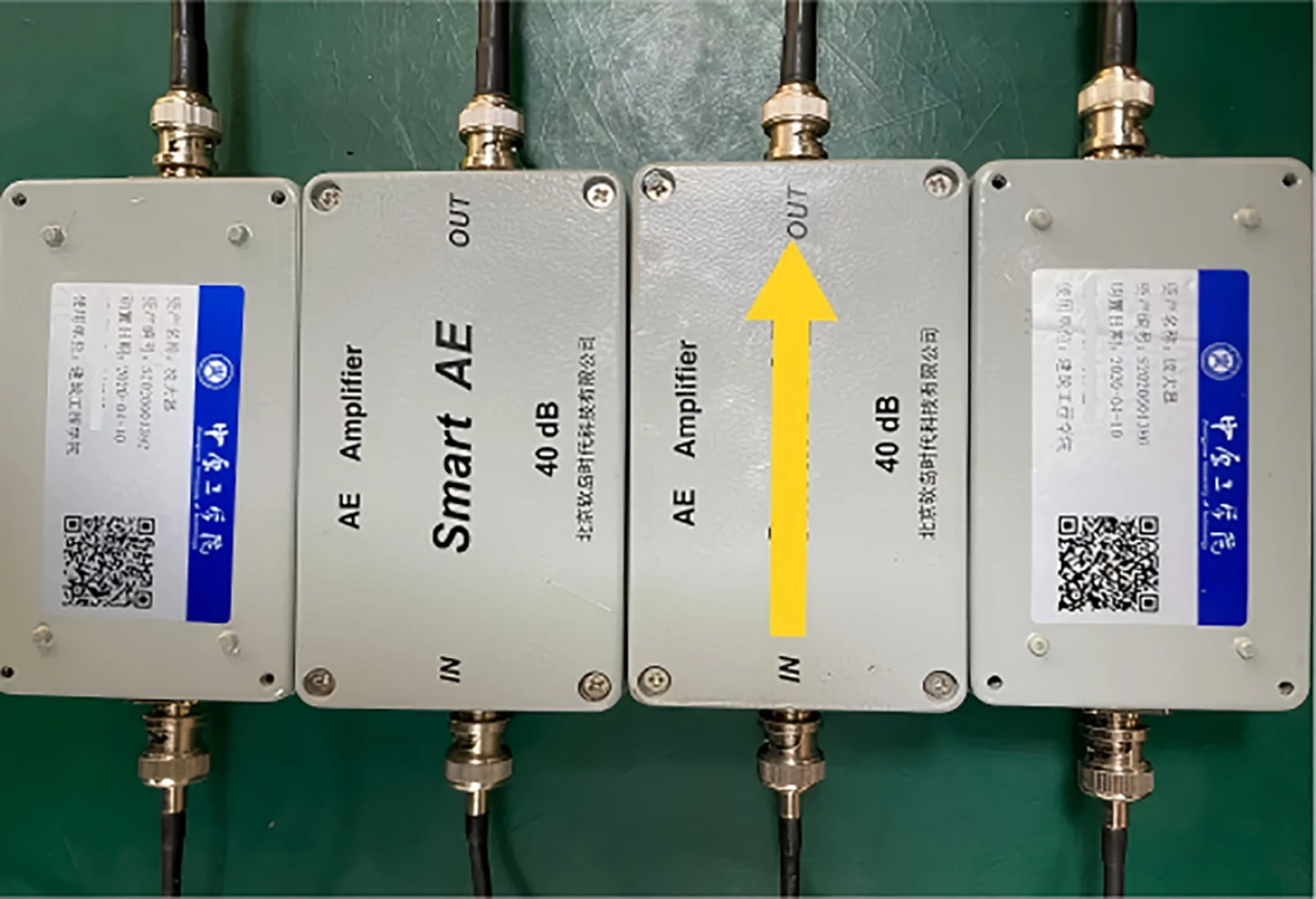
Signal amplifier.

To verify the effectiveness and positioning accuracy of the SEU-Net model in actual rock fracture microseismic monitoring [25], this section presents a sandstone uniaxial compression acoustic emission monitoring test. Three standard cubic sandstone specimens with dimensions of 150 mm × 150 mm × 150 mm were selected for the test, numbered 1#, 2#, and 3#. All specimens were taken from the same rock mass, and the processing accuracy met the standards recommended by the International Society for Rock Mechanics [26]. The detailed physical and mechanical parameters of the specimens are shown in Table 4. The physical layout of the loading system and acoustic emission signal acquisition system is shown in the figure (Figs 8–10).

**Table 4:** Basic physical and mechanical parameters of sandstone specimens.

Test Block Number	Weight (g)	Loading rate(mm/min)	P-wave velocity (m/s)	Loading rate (mm·min-1)	Size
1#	8537	0.3	3250	0.3	150mm × 150 mm × 150 mm
2#	8545	0.3	3250	0.3	150mm × 150 mm × 150 mm
3#	8528	0.3	3250	0.3	150mm × 150 mm × 150 mm

The experimental loading apparatus employed a YAW6206 microcomputer-controlled electro-hydraulic servo testing machine with a maximum load capacity of 2000 kN. To ensure that the specimens failed under quasi-static conditions and to completely record the entire fracture process, the experiment adopted a displacement control mode with a constant loading rate of 0.3 mm/min. The system automatically and synchronously recorded the load and displacement data [27].

**Fig 11:**
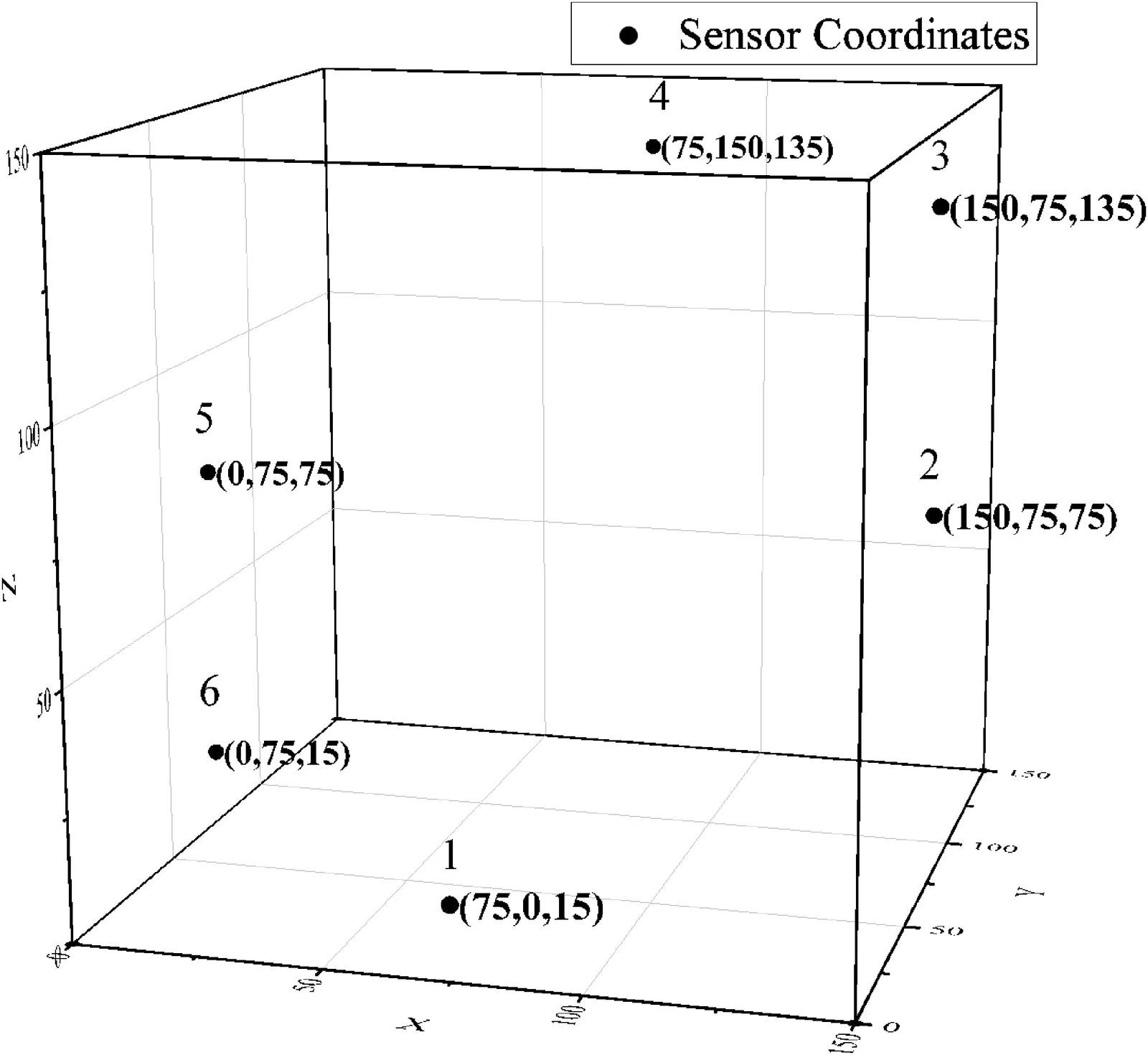
Sensor layout diagram.

An 8-channel acoustic emission monitoring system was used in the experiment. As shown in Fig 11, six detectors were arranged on the surface of the test block to form a spatial three-dimensional monitoring array. The three-dimensional spatial coordinates of each sensor were recorded. Coupling agent was used to ensure error-free signal transmission. The monitoring system parameters were set with a sampling frequency of 3 MHz and a preamplifier gain of 40 dB (see Fig 10). Before the experiment, a lead-breaking experiment was used to calculate the wave velocity of the test block [28]. By breaking pencil lead at preset points on the surface of the test block to simulate an acoustic emission source, the excited elastic wave signal was collected. The preset wave velocity was corrected by an iterative inversion algorithm until the deviation between the positioning result and the actual lead-breaking position was minimized, thereby determining the equivalent P-wave velocity of this batch of sandstone test blocks, providing reliable velocity model parameters for the accurate algorithmic positioning of subsequent microseismic events.

### 4.1. Data processing and analysis

**Fig 12:**
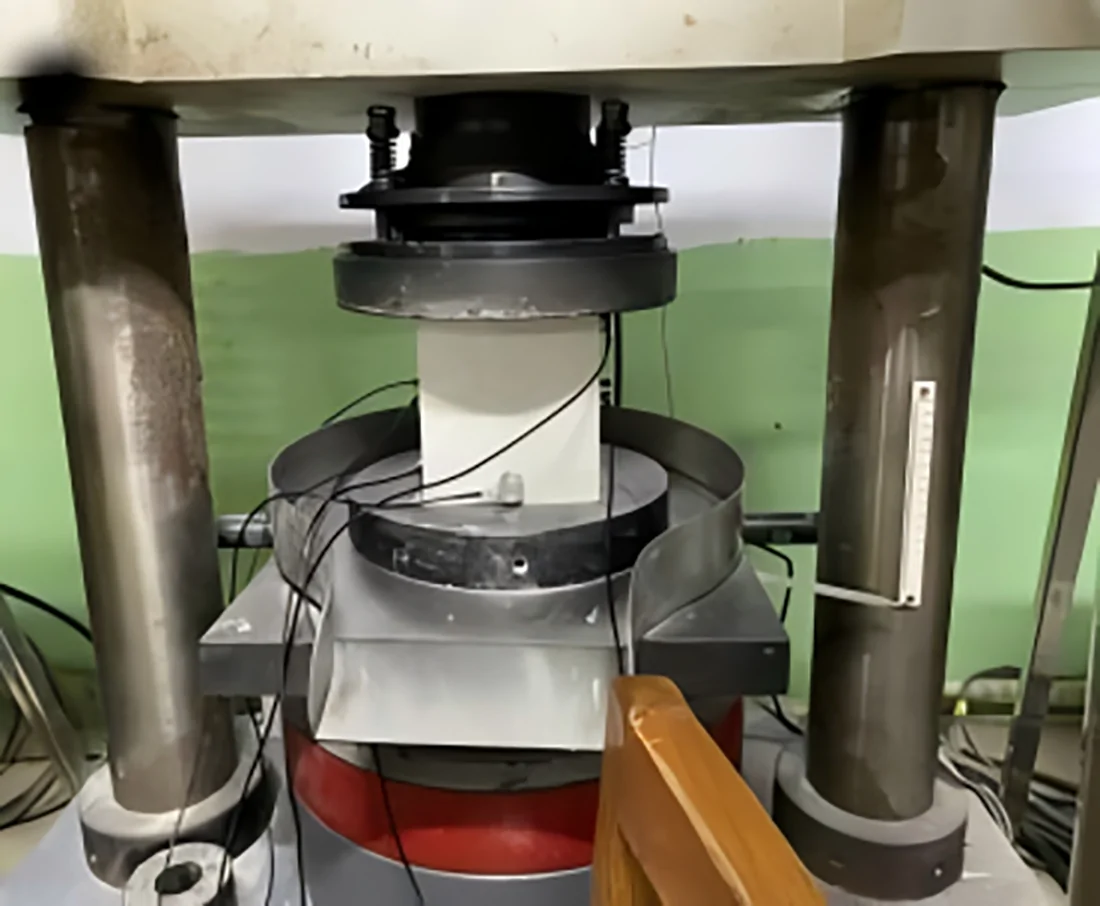
Fracturing preparation.

**Fig 13:**
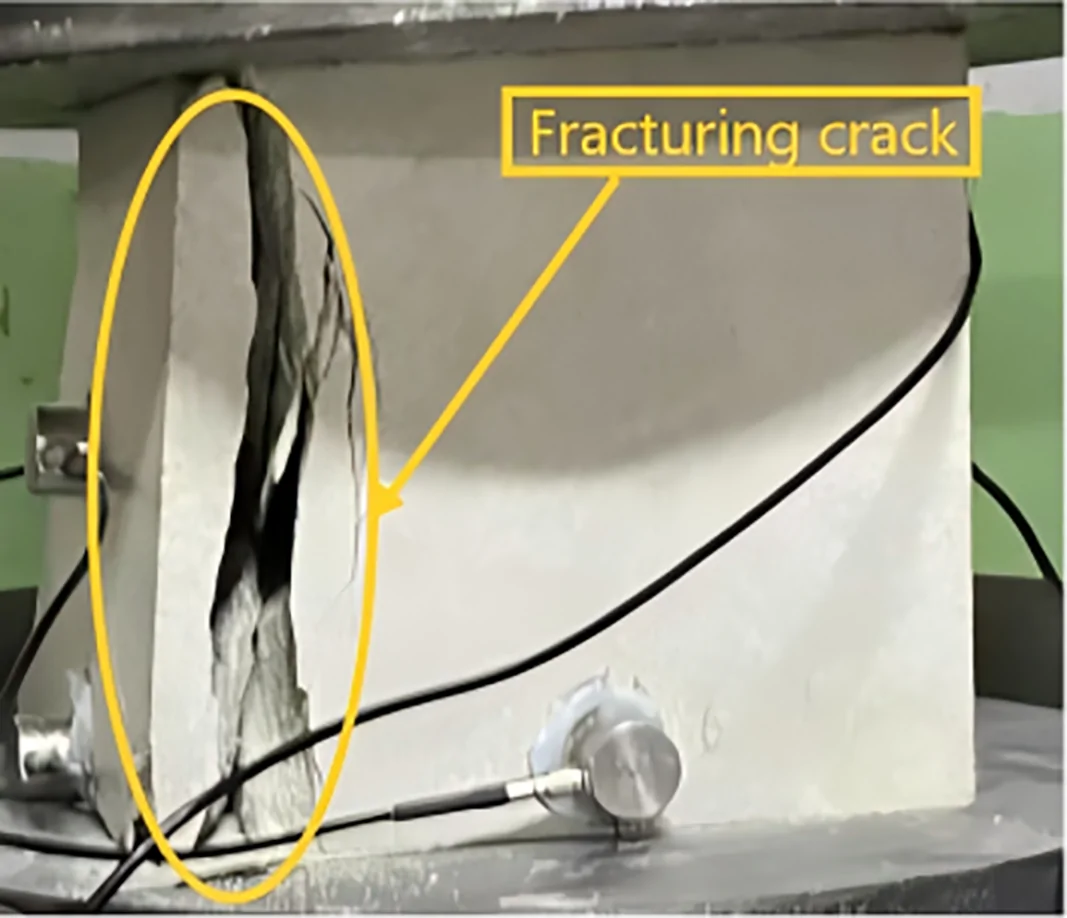
Crack.

To evaluate the localization accuracy and stability of different picking methods in practical engineering scenarios, this study employed the built-in acoustic emission software, the AIC method, the standard U-Net, and the proposed SEU-Net algorithm to process the microseismic events acquired during the experiment. Origin software was utilized to plot the 3D source spatial distribution maps, which were subsequently compared and analyzed against the actual fractured specimen. As shown in the photograph of the fractured specimen in Figs 12 and 13, the specimen exhibits compressive failure characteristics under uniaxial compression: a distinct through-going main crack appears on the ACEG face, and microcrack branches extending towards the upper right are visible in the middle of the specimen. These macroscopic physical features serve as the baseline for evaluating the localization accuracy of the respective algorithms.

**Fig 14:**
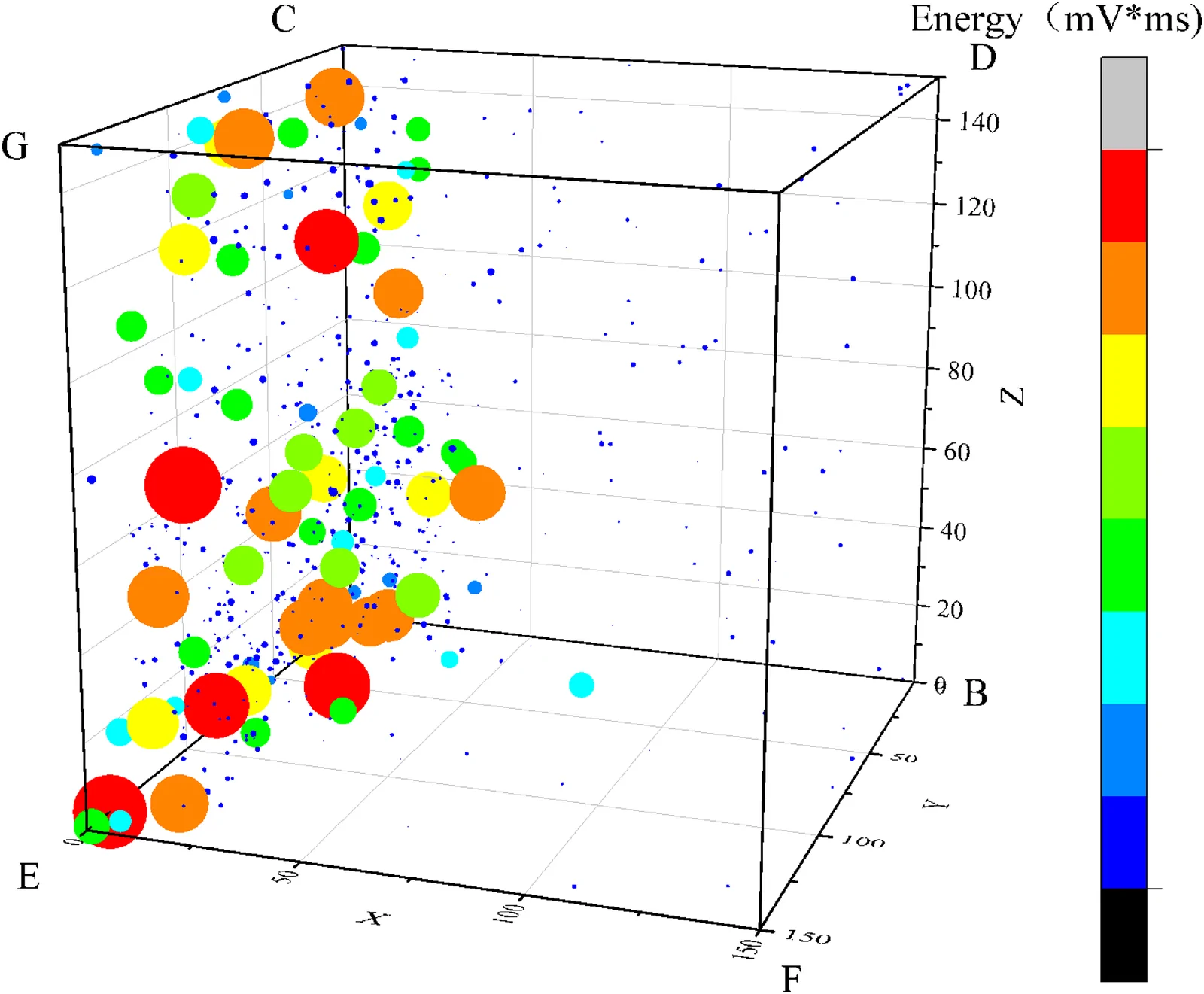
Acoustic emission software positioning energy map.

**Fig 15:**
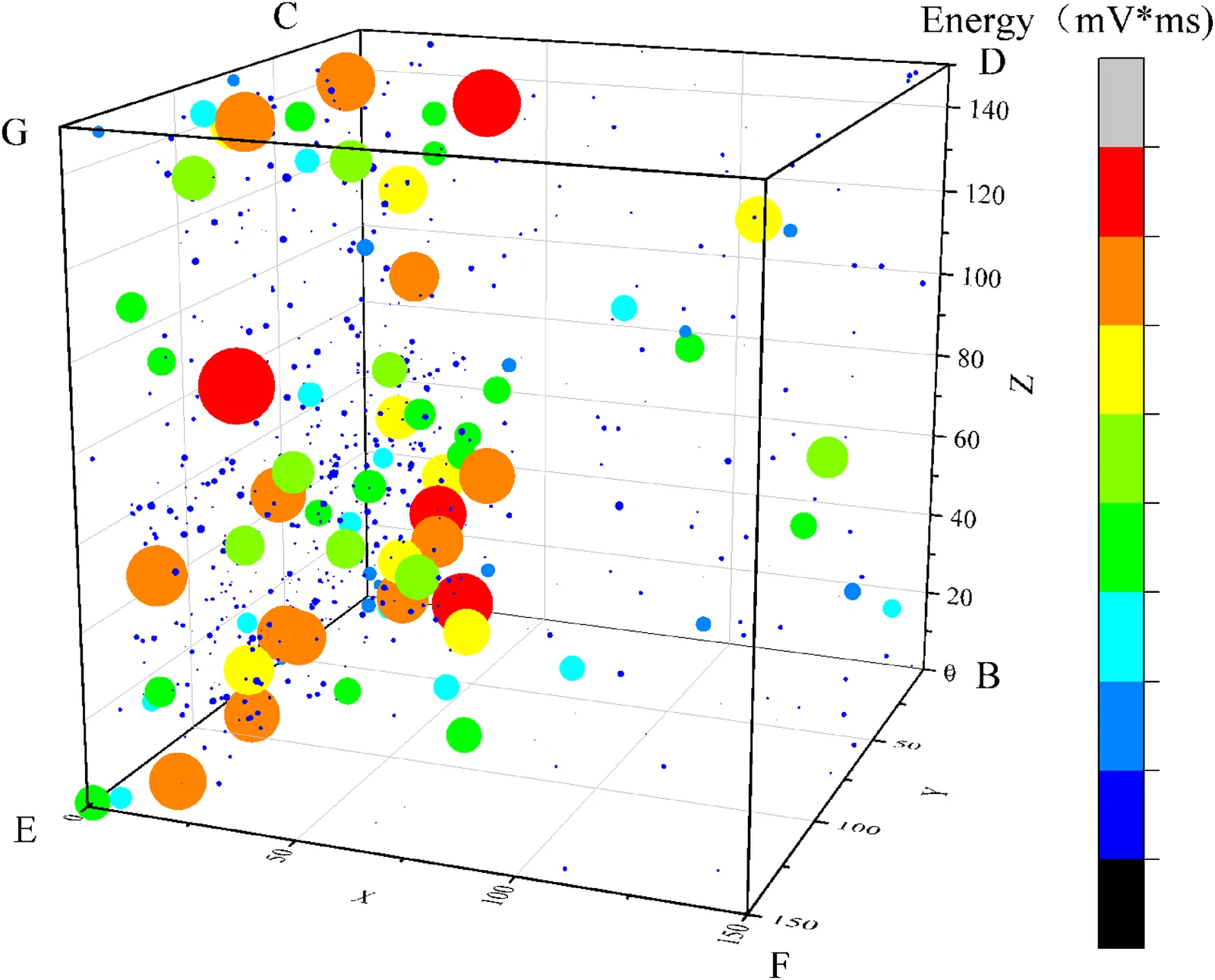
SEU-net location energy map.

**Fig 16:**
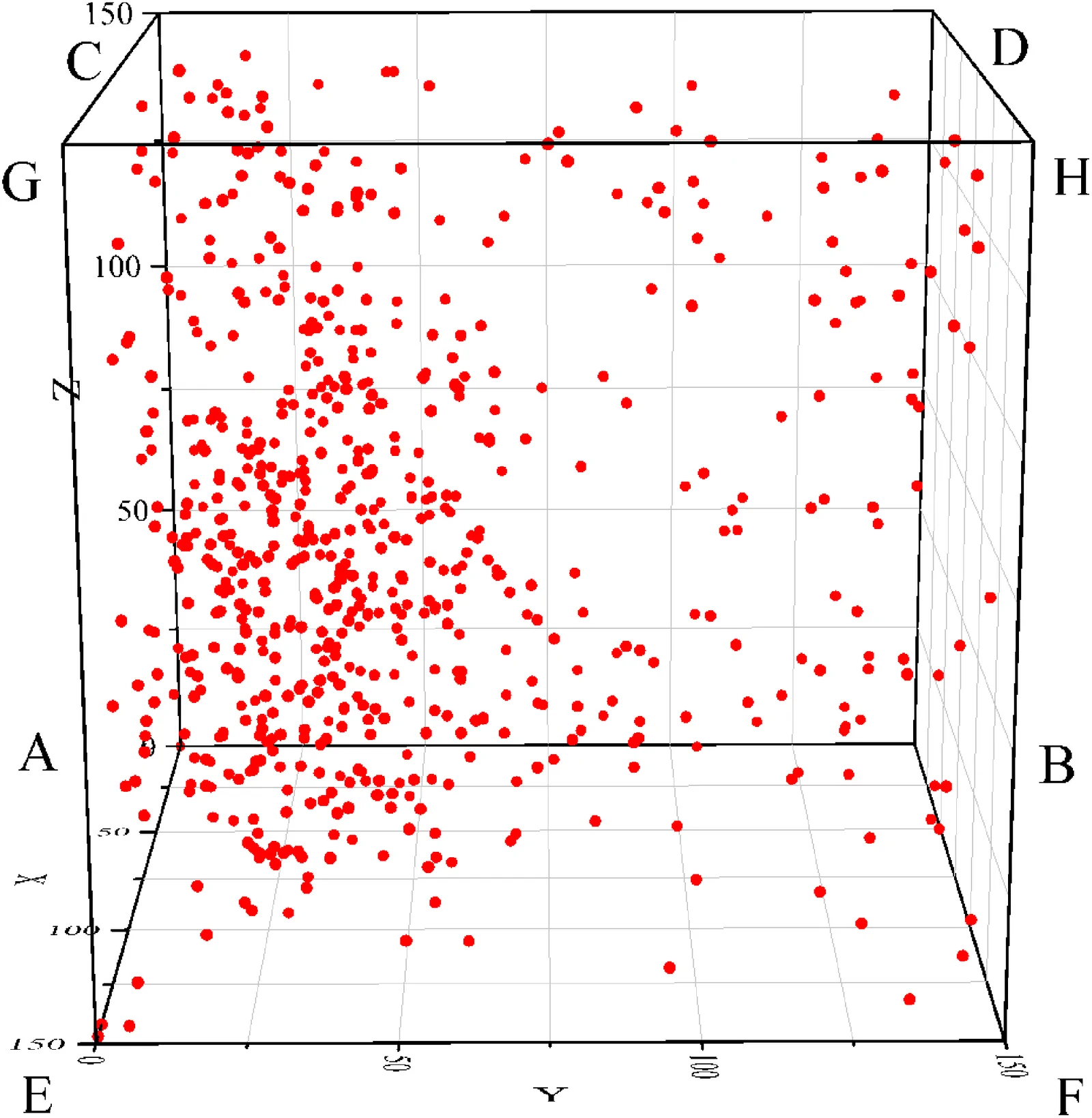
Acoustic emission software positioning.

**Fig 17:**
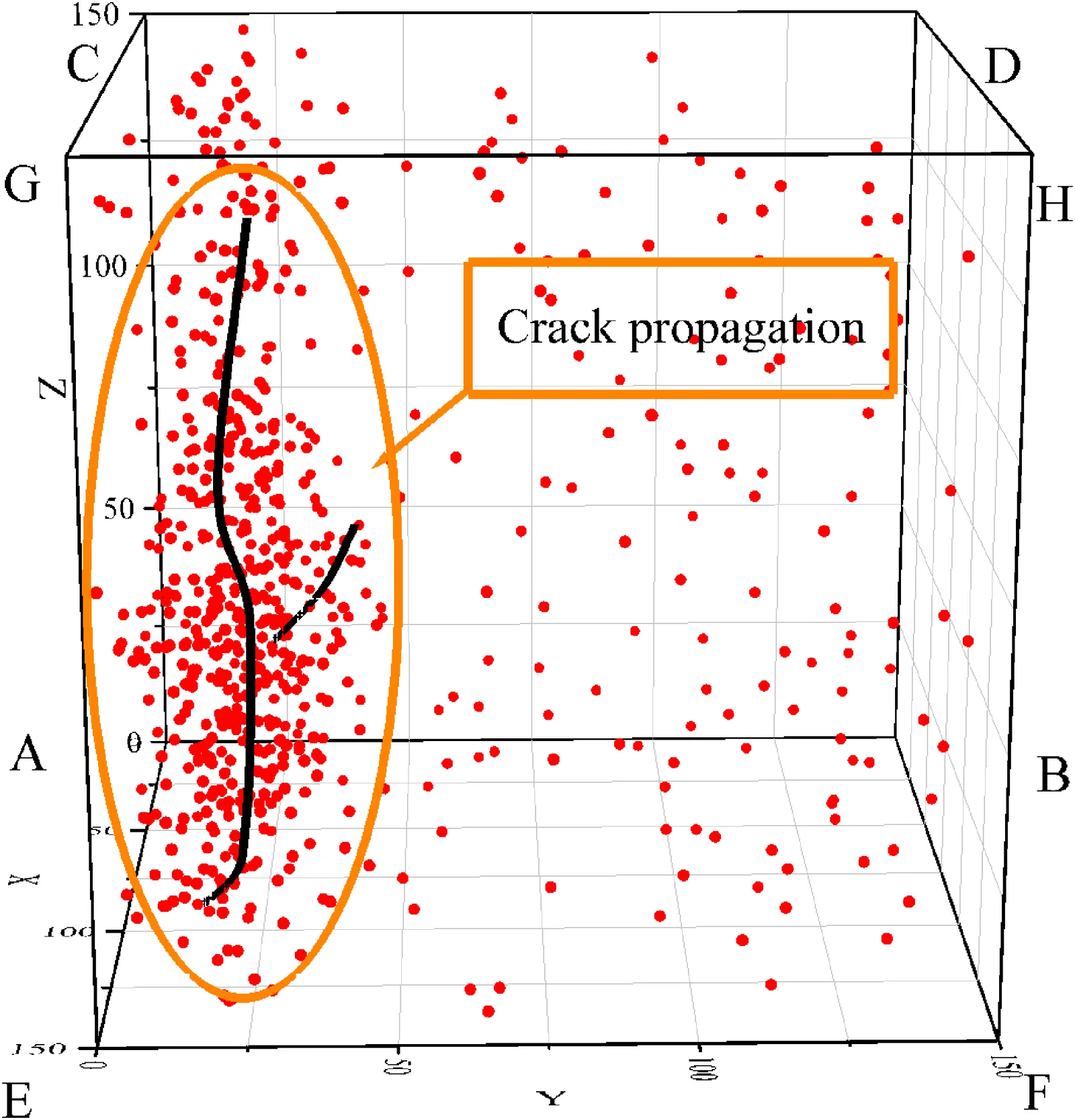
AIC picking event.

**Fig 18:**
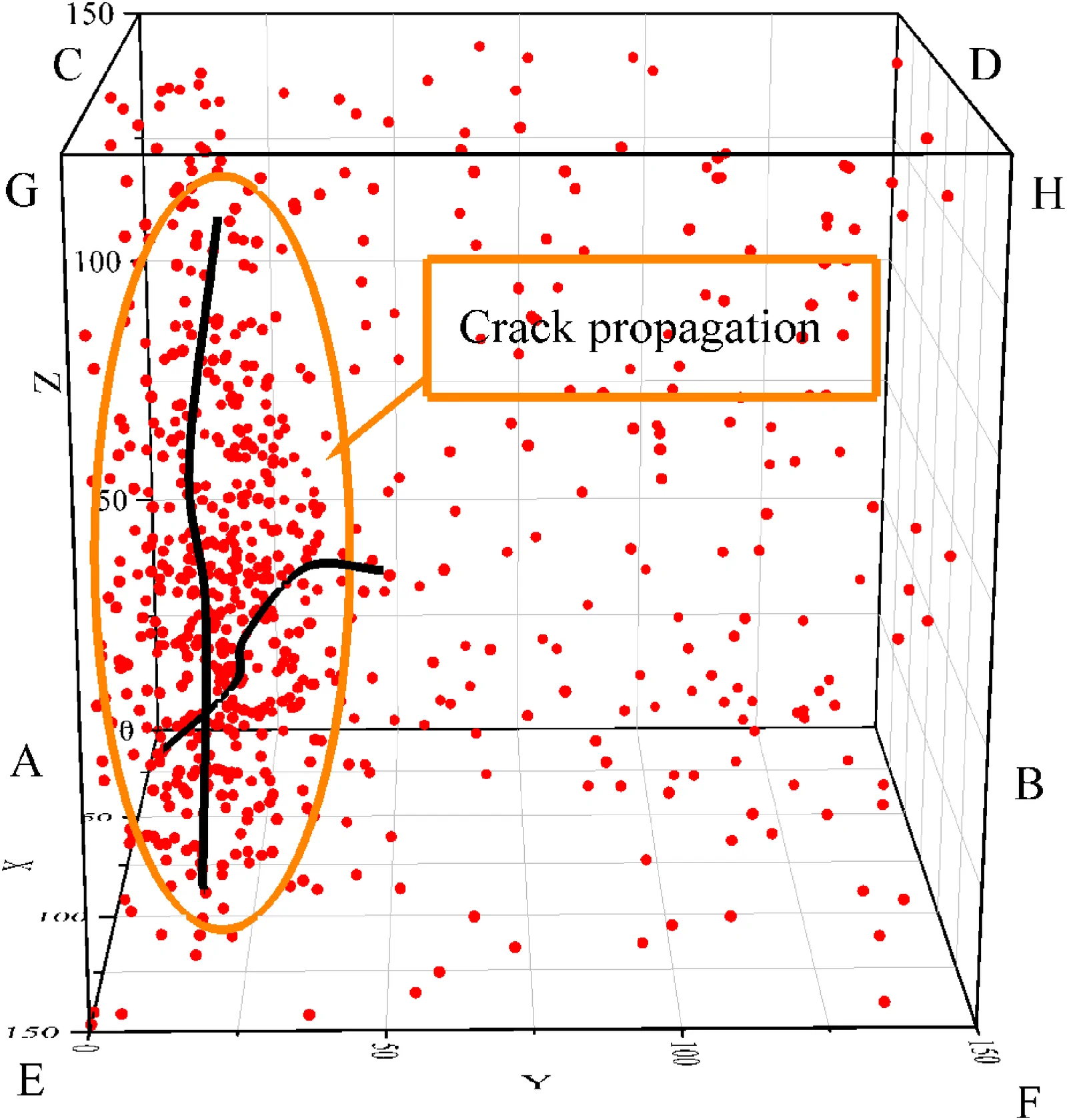
U-Net picks events.

**Fig 19:**
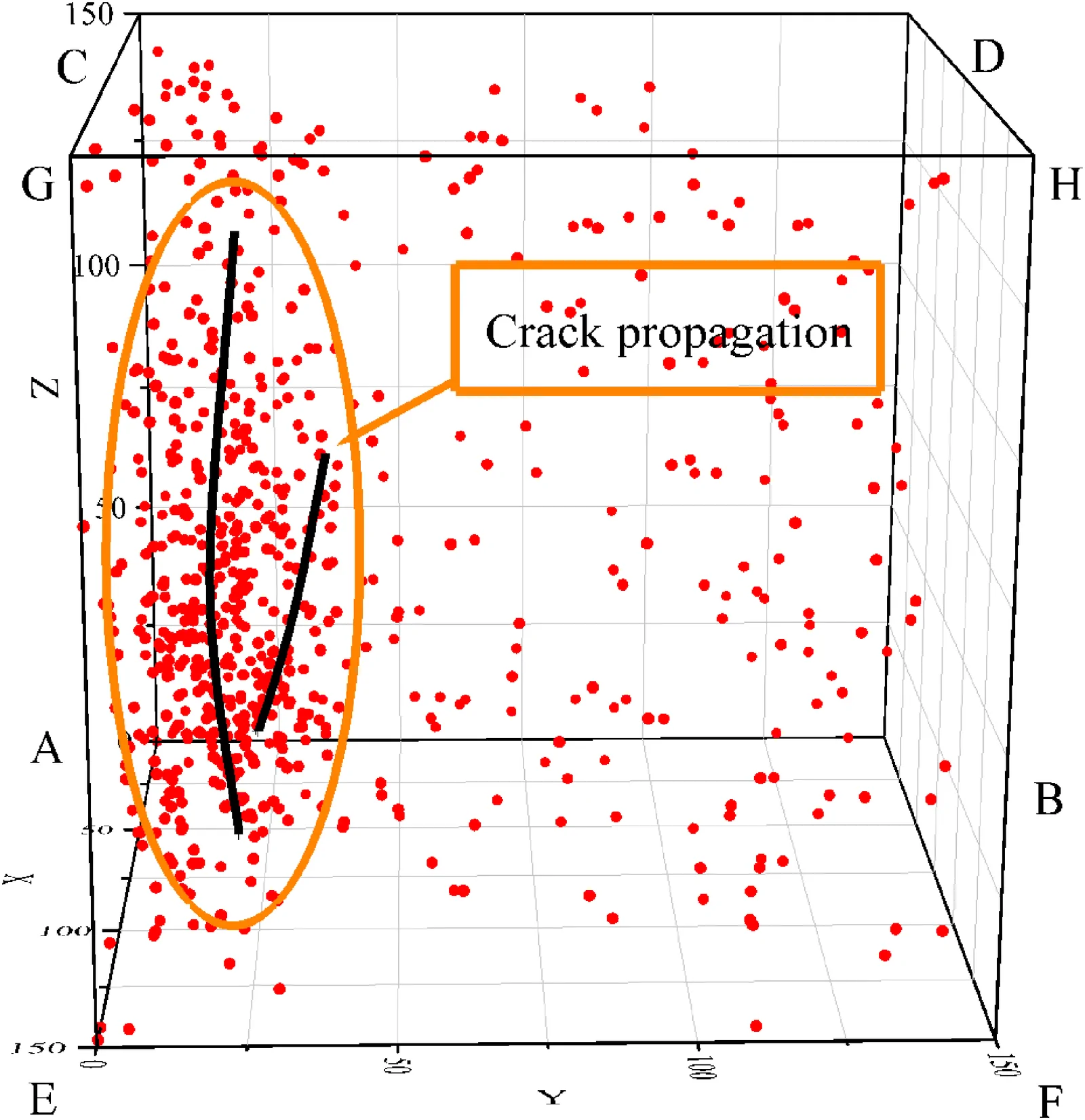
SEU-Net picking events.

Figs 14–16 respectively illustrate the spatial localization and energy distribution of events processed by the built-in acoustic emission software. The results show that the location event points are spatially dispersed, mainly concentrated in the lower left front region of the test block, failing to form a clear energy accumulation zone, indicating insufficient picking accuracy of the acoustic emission localization software. This results in the source distribution effect failing to reflect the actual evolution path.The localization results of the AIC method in Fig 17 and the standard U-Net in Fig 18 show significant improvements over those obtained by the built-in software. The events picked and localized by the AIC method are mainly distributed in the middle-lower region to the right of the EG line, with the top events corresponding well to the macroscopic vertical cracks. The localization results of U-Net exhibit a distribution pattern similar to that of the AIC method; the events are also concentrated to the right of the EG line and display a trend of crack propagation toward the right, which is fundamentally consistent with the macroscopic baseline feature of microcracks extending toward the upper right.Figs 15 and 19 display the energy distribution and localization results of the proposed SEU-Net model.Compared to the previous methods, the model’s location events exhibit better spatial clustering and continuity. High-energy events are densely distributed in the middle of the specimen and near the main fracture region, and are similar along the potential crack propagation direction. For the orientation of micro-branch cracks, the location map shows a banded trend.

In summary, the location results of AIC, U-Net, and SEU-Net are close to the actual fracture locations and can reflect the characteristics of the main crack to a certain extent. SEU-Net’s source distribution in the laboratory environment is even closer to the actual fracture crack, verifying the accuracy of this method in practical applications of microseismic monitoring.

## 5. Discussion

This paper addresses the challenges of insufficient accuracy and poor stability in microseismic signal acquisition under low signal-to-noise ratio environments. It proposes an automatic microseismic signal segmentation model based on the Squeeze-and-Excitation attention mechanism using SEU-Net. The main conclusions are as follows:

The SEU-Net model is proposed based on the U-Net encoder-decoder architecture. By embedding the SE attention module, the dependency relationship between feature channels is explicitly modeled, so as to achieve adaptive enhancement of effective features of microseismic signal mutation and suppression of background noise features. The one-dimensional time dimension is extended to solve the input format mismatch problem, and the advantages of multi-scale feature extraction of two-dimensional convolution are brought into play to enhance the representation ability.

SEU-Net exhibits excellent noise robustness and picking stability under low signal-to-noise ratio conditions. The model training process converges smoothly, with a test set picking accuracy of 98.97%. In tests with a signal-to-noise ratio of 5 dB, the model maintains picking accuracy of 79.98% and 89.93% for the start and end points of events, respectively, which is about 10 percentage points higher than the benchmark U-Net. Probability curve analysis shows that this mechanism effectively overcomes the feature lag and premature signal attenuation problems of traditional models under strong noise conditions.

In uniaxial compression tests of sandstone, the source location can be determined based on the results obtained using this method, which can accurately depict the evolution of damage within the rock mass. It has good engineering application value.

It should be noted that the data used in this study were derived from laboratory uniaxial compression acoustic emission tests. The acoustic emission signals obtained from these experiments are characterized by a higher frequency band, relatively simple noise components, and controllable propagation paths. In contrast, field microseismic signals in mining and geotechnical engineering environments are jointly influenced by complex geological structures, non-stationary background noise, and mechanical noise, resulting in waveform characteristics that exhibit significant complexity and non-stationarity. Based on a controlled laboratory environment, this work aimed to validate the fundamental methodology. It confirmed the feasibility and effectiveness of the proposed SEU-Net model for signal segmentation and first-arrival detection under low signal-to-noise ratio (Low-SNR) conditions, laying the technical foundation for subsequent engineering implementation.

To further enhance the engineering applicability of this method, subsequent research will focus on the following tasks: First, establishing a transfer learning mechanism tailored to engineering scenarios by collecting microseismic monitoring data from mine sites and fine-tuning pre-trained models to adapt to the complex characteristics of field signals; Second, conducting systematic field trials to verify the model’s detection stability and source localization accuracy in real-world, complex noise environments; third, exploring joint inversion and intelligent processing techniques for multi-channel, multi-component data to advance the large-scale and intelligent application of this technology in complex engineering sites.
